# Biomimetic 3D Prototyping of Hierarchically Porous Multilayered Membranes for Enhanced Oil–Water Filtration

**DOI:** 10.1021/acsami.4c18528

**Published:** 2025-01-23

**Authors:** Abhishek Saji Kumar, Rayane Akoumeh, Arunachalam Ramanathan, JaeWoo Park, Varunkumar Thippanna, Dhanush Patil, Yuxiang Zhu, Dharneedar Ravichandran, Sri Vaishnavi Thummalapalli, M. Taylor Sobczak, Lindsay Bick Chambers, Taylor G. Theobald, Churan Yu, Chao Sui, Libin Yang, Deepalekshmi Ponnamma, Mohammad K. Hassan, Maryam Al-Ejji, Sui Yang, Kenan Song

**Affiliations:** † Materials Science and Engineering, School for Engineering of Matter, Transport and Energy (SEMTE), Ira A. Fulton Schools of Engineering, Arizona State University (ASU), Tempe, Arizona 85281, United States; ‡ Center for Advanced Materials, Qatar University, P.O. Box 2713, Doha, Qatar; § Mechanical Engineering, College of Engineering, University of Georgia, 302 E. Campus Rd, Athens, Georgia 30602, United States; ∥ School of Manufacturing Systems and Networks (MSN), Ira A. Fulton Schools of Engineering, Arizona State University (ASU), Mesa, Arizona 85212, United States; ⊥ Department of Mechanical Engineering, University of California, Berkeley, California 94720, United States; # Center for Molecular Design and Biomimetics at the Biodesign Institute, Arizona State University, Tempe, Arizona 85281, United States; ∇ Associate Professor of Mechanical Engineering, College of Engineering, University of Georgia (UGA), 302 E. Campus Rd., Athens 30602, United States; ○ Adjunct professor at the School of Manufacturing Systems and Networks (MSN), Ira A. Fulton Schools of Engineering, Arizona State University (ASU), Mesa, Arizona 85212, United States

**Keywords:** 3D printing, multilayer membrane, oil−water filtration, antifouling, additive manufacturing, PVDF, hierarchical structure, scalable

## Abstract

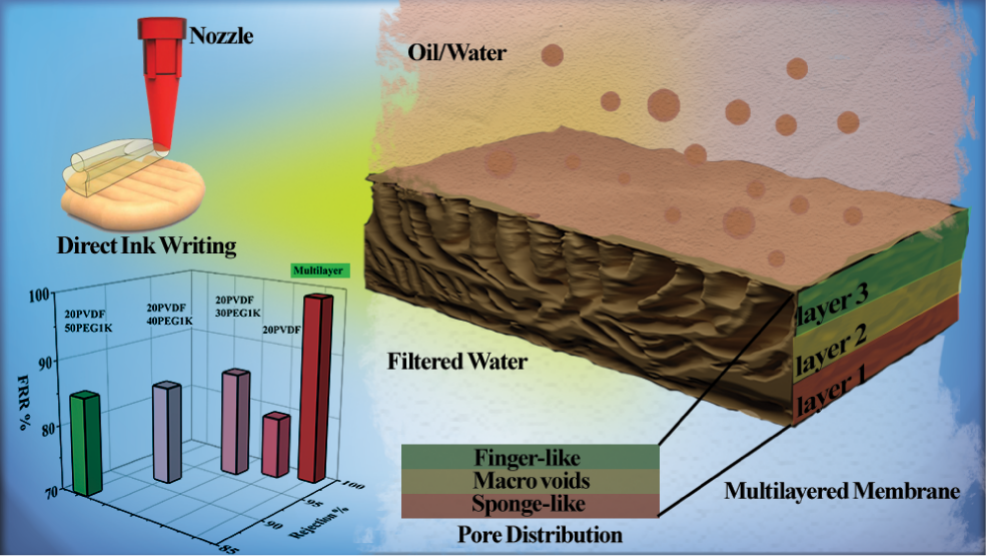

This study introduces a biomimetic approach to 3D printing multilayered hierarchical porous membranes (MHMs) using Direct Ink Writing (DIW) technology. Fabricated through a fast layer-by-layer printing process with varying concentrations of pore-forming agents, the produced MHMs mimic the hierarchical pore structure and filtration capabilities of natural soil systems. As a result, the 3D-printed MHMs achieved an impressive oil rejection rate of 99.02% and demonstrated exceptional reusability, maintaining a flux recovery ratio of 99.48% even after hours of continuous filtration. Moreover, the 3D-printed MHMs exhibit superior hierarchical porous architecture and mechanical integrity compared to traditional flat sheet single-layered membranes. This study presents a significant advancement for scalable 3D printing of customized multilayer membranes with tailored porosity and high-performance filtration properties. The simplicity, versatility, and cost-effectiveness of the presented manufacturing method offer a pathway for advanced design and on-demand membrane production.

## 1 Introduction

The increasing discharge of industrial wastewater and frequent offshore oil spills have posed significant threats to the global environment and public health.^1−5^ Many countries, for instance, with vast oil reserves, face heightened risks of oil spills due to intensive human activities, resulting in far-reaching consequences that impact air quality, marine and terrestrial ecosystems, and human health.^6,7^ Additionally, climate change and escalating global water consumption have compounded these issues, with most used water being inadequately treated and discharged back into freshwater sources, further exacerbating water pollution.^8,9^ These challenges underscore the need for advanced materials capable of efficiently treating oil–water emulsions and removing contaminants from municipal and industrial wastewater.^10−12^

Current methods for oily wastewater treatment primarily involve chemical and physical separation techniques. Chemical methods, such as de-emulsification, coagulation, and flocculation, and physical methods, including gravity separation, adsorption, skimming, and dissolved air flotation, are widely employed.^13−16^ However, these approaches suffer from limitations such as high toxicity, additional waste generation, and difficulty in meeting stringent environmental regulations.^17−19^ The reliance on costly, infrastructure-heavy equipment further hampers their efficiency, often resulting in inadequate water treatment. As a result, porous membrane technology has emerged as a robust alternative, offering simplicity, high rejection rates, and the potential for continuous operation without drawbacks via traditional methods.^20^

Porous membrane fabrication is typically achieved through techniques like solvent-casting phase inversion,^21,22^ foaming,^23^ particulate leaching,^24^ stretching,^25^ electrospinning,^26^ and immersion precipitation.^27−29^ However, these conventional methods often require additional post-treatments, such as vacuum filtration or nanoparticle incorporation and coatings,^30^ to fine-tune membrane porosity and wettability.^31^ The extensive use of solvents/coagulants not only increases costs but also poses environmental risks, and the membranes produced often face a trade-off between selectivity and permeability, as well as susceptibility to fouling—major obstacles to their large-scale application.^32−35^ Additive Manufacturing (AM), particularly 3D printing, offers a transformative approach to membrane fabrication, combining cost-effectiveness, precision, and scalability with the ability to control pore size and uniformity.^36−38^ While the overarching goal remains the provision of clean water, the methodologies to achieve this, particularly those leveraging AM techniques such as the fabrication of biomimetic structures are still in their nascent stages and face significant challenges in real-world applications.^39,40^ These structures, although promising, often lack the robustness and scalability required for practical deployment. Moreover, when scaling up production, material efficiency becomes a critical factor, with many of the current approaches exhibiting high levels of material wastage, thereby limiting their feasibility for large-scale implementation.^41,42^ Addressing these limitations is imperative to bridge the gap between laboratory innovation and sustainable industrial application. Direct Ink Writing (DIW), a versatile extrusion-based AM technique, enables the printing of viscous inks and offers a straightforward setup integrated with 3D printers, allowing for rapid and precise membrane fabrication. DIW’s printing speeds, ranging from 500 to 3100 mm/s,^43,44^ surpass those of conventional membrane manufacturing processes, highlighting its potential as a swift and adaptable fabrication method. However, most of the studies have focused on creating single-layered microfiltration membranes due to the ease of control on thicknesses and pores.^45^ Still, to achieve simultaneously thin, multilayered, hierarchical porous membranes for efficient water treatment remains challenging.

This study presents a novel approach to fabricating multilayered hierarchical membranes (MHMs) using DIW 3D printing, combining polyvinylidene fluoride (PVDF)^46,47^ with polyethylene glycol (PEG) as a porogen to enhance efficiency in oil–water separation. The innovative DIW process allows for precise control over each membrane layer’s thickness, achieving thin-film membranes within the 150 μm range using pneumatic or electric fluid dispensers and fine nozzles to regulate fluid deposition. By adjusting PEG concentrations (30 to 50 wt %) across different layers, the resulting membranes form a hierarchical porous structure that mimics natural sand aggregates, providing enhanced rejection capabilities. This research offers a comprehensive analysis of the printing parameters, PEG’s effects, and the resulting membrane characteristics, demonstrating their high performance in oil–water separation applications. The integration of DIW technology with PVDF–PEG blends provides a scalable pathway for tailored membrane design, potentially revolutionizing point-of-use (POU) water filtration systems. These advancements hold significant promise for addressing water scarcity challenges in regions like Bangladesh,^48^ South Africa,^49^ and Gulf countries,^50^ where efficient and sustainable water treatment solutions are urgently needed.

## 2 Experimental Section

### 2.1 Materials and Reagents

Polyvinylidene fluoride (PVDF) (Solef 1015) with a molecular weight (Mw) of 570 kDa was obtained from Solvay Specialty. The pore-forming agents, polyethylene glycol 600 (PEG600), with an average Mw of 570–630 g/mol, and PEG 1000 (noted as PEG1K) with an average Mw of 950–1050 g/mol, both with CAS# 25322-68-3, along with dimethylacetamide (DMAc), ≥ 99.8%, CAS# 127-19-5, were sourced from Sigma-Aldrich and used without further modification as inks for 3D-printed membrane preparation. For oil–water emulsion preparation, crude oil (internal standard) and sodium dodecyl sulfate (SDS) (CAS# 151-21-3) from Sigma-Aldrich were used as received without any modifications.

### 2.2 Ink Formulation and 3D Printing Membrane

The inks were formulated to meet the shear-thinning requirements for DIW 3D printing. A solution containing 20 wt % PVDF and varying PEG concentrations (30, 40, and 50 wt %) were prepared by dissolving the components in DMAc and stirring continuously at 150 rpm for 12 h at 90 °C. Upon complete dissolution, the solution was transferred to a vacuum chamber to remove any air bubbles introduced during stirring. The bubble-free solution was then carefully loaded into DIW syringes, taking care to avoid the introduction of air bubbles that could lead to voids during the printing process. A Hyrel SR printer was used for membrane fabrication, featuring a large build volume of 200 mm in the x, y, and z directions and a positional resolution of 1 μm in the *z*-direction. Printing began with the nozzle following predefined paths set by G-codes, extruding the ink at a speed of 40 mm/s with a layer height of 0.15 mm. After printing, the glass substrate with the printed membrane was immersed in deionized (DI) water for 30 min to induce phase inversion. The resulting membranes were stored in DI water for subsequent experiments. See the samples and their specific compositions in Table 1.

**Table 1:** Nomenclature of the Prepared Membrane Samples from 3D Printing, Their Composition, and Their Microstructural Features^a^

	Membrane compositions			3D printing				
Samples	PVDF/solvent (wt %)	PEG 600/PVDF (wt %)	PEG1K/PVDF (wt %)	T (°C)	P (bar)	Speed (mm/s)	Nozzle diameter (mm)	t (μm)
15PVDF	15	-	-	90	1	40	0.21	120 ± 10
20PVDF	20	-	-				0.41	150 ± 10
20PVDF30PEG600		30	-					160 ± 10
20PVDF40PEG600		40	-					160 ± 10
20PVDF50PEG600		50	-					150 ± 10
20PVDF30PEG1K		-	30				0.26	160 ± 10
20PVDF40PEG1K		-	40					150 ± 10
20PVDF50PEG1K		-	50					160 ± 10
Multilayered		-	30/40/50					250 ± 5

a T: temperature; P: pressure; t: thickness.

### 2.3 Preparation of Oil–Water Emulsion

The oil–water emulsion was prepared using a typical procedure in which 30 mg of sodium dodecyl sulfate (SDS) was introduced into 500 mL DI water. Next, 0.18 mg of crude oil was added to the mixture and agitated using a mechanical stirrer for 30 min at 1000 rpm, followed by sonication for an additional 30 min. The solution was then transferred to a separatory funnel and allowed to settle for 4 h to facilitate the removal of any free oil layer. The resulting homogeneous solution was transferred to a glass container and stored in a refrigerator at 4 °C for further experiments.^51^

### 2.4 Membrane Characterization and Performance Evaluation

#### 2.4.1 Membrane Printability

The printability of the membranes was assessed through a comprehensive analysis of the viscosity of each solution. Viscosity measurements were conducted using a 25 mm parallel plate rheometer with a Peltier plate (Discover Hybrid Rheometer HR2, TA Instruments). The viscoelastic properties of the PVDF–PEG blends were evaluated at room temperature, covering a shear rate range from 0.1 to 10,000/s.

#### 2.4.2 Membrane Morphology

The membrane structure was examined using a Zeiss Auriga scanning electron microscope (SEM) operating at a voltage range of 5 kV to 20 kV. Prior to analysis, a thin layer of gold was sputter-coated onto the membrane surface to enhance conductivity. Surface porosity and cross-sectional images were analyzed using ImageJ software. Surface roughness was characterized using an atomic force microscope (AFM) MFP-3D system (Asylum Research, USA). The surface area and pore volume were determined through adsorption–desorption isotherms using the Brunauer–Emmett–Teller (BET) method, conducted with a Micromeritics ASAP 2420 system, using Silica–Alumina with a multipoint surface area of 198 ± 6 m^2^/g and a total pore volume of 0.61 ± 0.08 cm^3^/g as reference material. All samples were dried at 105 °C for 2 h before testing. The membrane’s surface wettability was assessed using a sessile drop (SD) instrument (DataPhysics Instruments, Germany).

#### 2.4.3 Chemical Composition and Mechanical Properties

The elemental surface analysis was done by using an X-ray Photoelectron Spectrometer, Axis Ultra DLD (XPS). The absorption was recorded using a Fourier Transform Infrared Spectroscopy FTIR Perkin for a wavelength range of 500 to 4000 cm^–1^ at room temperature. X-ray diffraction (XRD) was used to study crystallography and the material’s physical properties, and the measurements were done using PANalytical EMPYREAN (Cu–Kα radiation, 1.54060 [Å]). The membrane’s mechanical properties were tested using the LLOYD instrument from AMETEK.

#### 2.4.4 Membrane Performance

An Amicon-stirred cell equipped with a magnetic stirrer operating at 200 rpm was used for all the filtration studies. A nitrogen cylinder was connected to the cell to maintain a constant operating pressure of 1 bar. DI water was used for the determination of pure water flux following eq 1:^52^
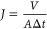
1

Where *J* is the pure water flux (L/m^2^·h), *V* represents the permeate volume (L), *A* denotes the effective filtration area (m^2^), and Δ*t* is the duration in minutes.

#### 2.4.5 Flux Recovery Ratio

The membrane’s initial pure water flux was assigned as *J*_0_. Then, the membrane was used to filter the produced water solution until the flux was reduced significantly, indicating fouling. The fouled membrane was then rinsed and backwashed for 20 min to remove the particles from the surface before being used again for filtration. The flux after backwash cleaning is noted as *J*_*i*_. Thus, the flux recovery ratio (FRR) was evaluated.^53^
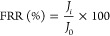
2

The Rejection *R (%)* was evaluated using a Nanodrop one spectrophotometer^53^ (Thermo Fisher Scientific).
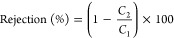
3where *C*_1_ and *C*_2_ are the concentrations of feed and filtrate solutions, respectively.

## 3 Results and Discussion

### 3.1 Multilayered Membrane Printability

**Figure 1:**
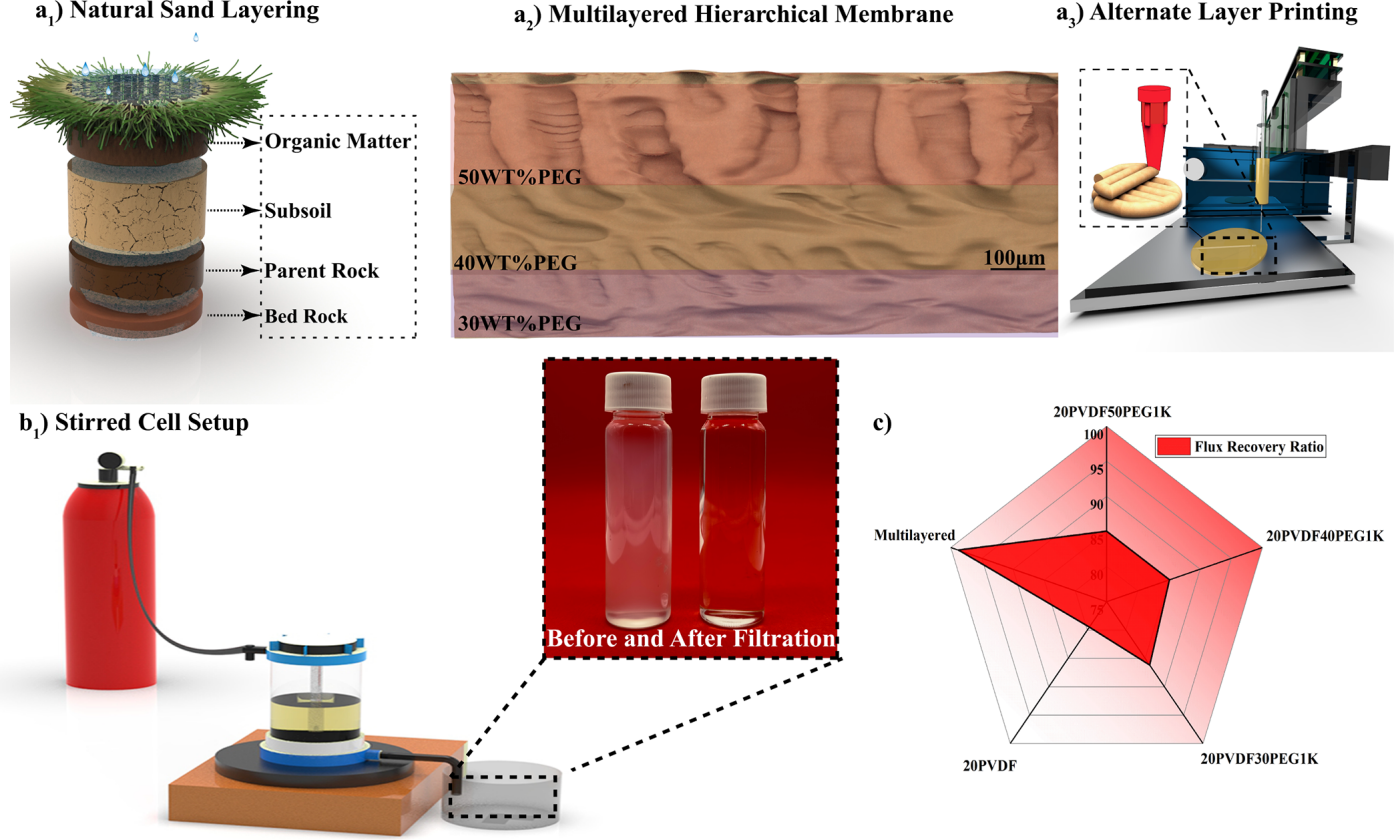
(a_1_) Illustration of the natural sand layering system, depicting the natural filtration process through distinct layers: organic matter, subsoil, parent rock, and bedrock, each with varying pore sizes and permeabilities. (a_2_) Cross-sectional view of the MHM showing layers with varying PEG (polyethylene glycol) concentrations (50, 40, and 30 wt %, see Table 1). (a_3_) Schematic of alternate layer printing using a 3D printer, demonstrating the fabrication process of multilayered hierarchical porous membranes. (b_1_) Diagram of the stirred cell setup used for the filtration of oil–water emulsions, highlighting the before and after filtration outcomes using MHMs. (c) Radar chart displaying the flux recovery ratio (FRR) of different 3D-printed membranes, emphasizing the superior performance of the multilayered membrane compared to other compositions.

**Figure 2:**
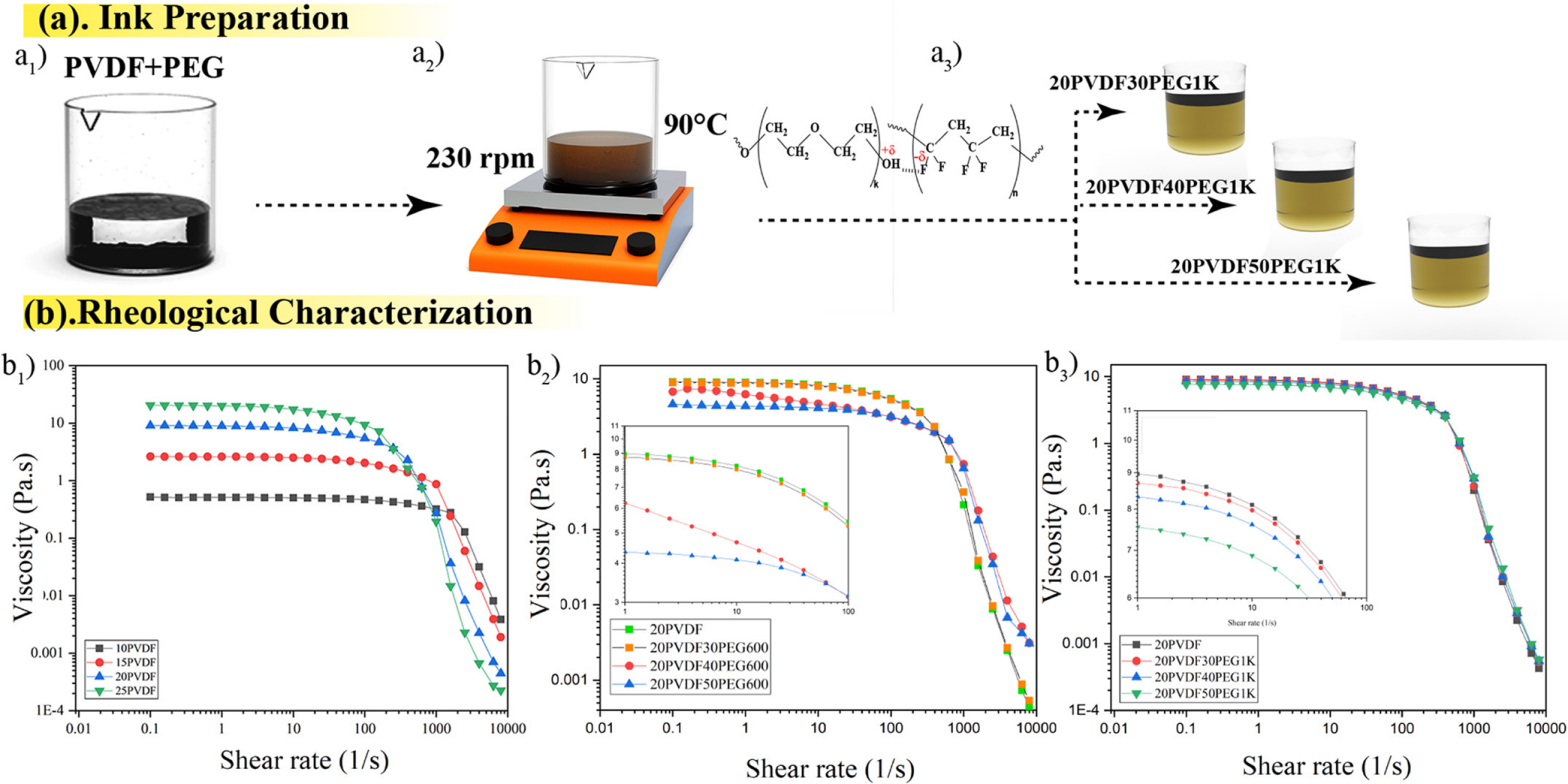
(a_1_–a_3_) Illustration of the ink preparation process showing the blending of PVDF with PEG, highlighting the formation of hydrogen bonds between PVDF and PEG during mixing at 230 rpm and 90 °C. The resulting formulations include different PVDF–PEG blends: 20PVDF30PEG1K, 20PVDF40PEG1K, and 20PVDF50PEG1K (see specific compositions in Table 1). (b_1_–b_3_) Rheological characterization graphs showing the viscosity versus shear rate for pure PVDF and PVDF–PEG blends containing PEG600 and PEG1K, demonstrating the shear-thinning behavior and the effect of PEG content on the rheological properties of the inks.

Addressing the critical need for effective oil removal due to oil spills and industrial accidents requires innovative membrane systems that are efficient, cost-effective, and capable of rapid deployment. In response, we have developed 3D-printed Multilayered Hierarchical Membranes (MHMs), as illustrated in Figure 1a_1_,a_2_, which mimic the highly efficient natural soil filtration processes. The natural sand layering system serves as an inspiration for the membrane design due to its efficient filtration process, where each distinct layer—organic matter, subsoil, parent rock, and bedrock—plays a specific role in trapping and filtering impurities through varying pore sizes and permeabilities. This concept is mimicked in the multilayered membrane design, where different polymer compositions and structural configurations are strategically used to replicate the hierarchical filtration capabilities of natural sand, enhancing the separation performance and stability of the synthetic membrane. These advanced membranes were fabricated using DIW (Figure 2a_3_), a 3D printing technique that stands out among additive manufacturing methods due to its ability to produce intricate and customizable “green bodies” that undergo postprocessing to form the final membrane structure. The DIW system’s configuration is pivotal in achieving the optimal balance of thinness and performance, enabling the production of membranes with precise microstructures and enhanced rejection rates. With DIW, membranes as thin as 60 μm can be printed,^44^ offering significant advantages over conventional techniques, such as tailored pore architectures that improve filtration efficiency and minimize fouling (see Table S1). The broader impact of these 3D-printed membranes lies in their ability to be rapidly manufactured and customized for specific separation tasks, offering a sustainable solution for environmental protection and industrial wastewater management. This approach not only advances the field of membrane technology but also provides a scalable and adaptable tool for tackling pressing environmental challenges worldwide.

To enable seamless multilayer printing, the DIW syringes are equipped with tapered needles and connected to a fluid dispenser, ensuring consistent flow and uniform deposition on the glass substrate throughout the printing process (Figure 1a_3_). A circular model with an 8 cm diameter was meticulously designed in SolidWorks to integrate seamlessly with the Amicon stirred cell (200 mL) for membrane fabrication. To achieve precise 3D printing, three-layered G-codes were initially generated with nozzle parameters optimized for an infill density of 100% as shown in Figure S1a. However, recognizing the limitations imposed by the inherent low viscosity of the ink—such as coalescence during deposition, which led to surface waviness—the G-codes were strategically modified. Adjustments focused on refining the interline spacing and regulating the printing speed to 40 mm/s. This systematic optimization minimized meniscus contraction and ensured controlled coalescence with preceding layers, enabling the formation of a consistently smooth and uniform membrane.^54,55^ These critical refinements not only enhanced the structural integrity of the membranes but also demonstrated the potential for precise customization in additive manufacturing techniques tailored to advanced filtration systems. Printing specifications, including layer height, nozzle thickness, and print speed, were programmed using Repetrel software. Optimizing parameters for printing single-layered membranes while maintaining a thickness of less than 150 μm shown in Figure S1b proved challenging due to variations in PVDF–PEG compositions during each print. As a result, a fluid dispenser was preferred over a mechanical syringe pump, offering superior control and resolution in the membrane printing process.^56^

Optimal solution formulation, nozzle selection, and pressure settings are crucial for the precise printing of membranes. Initially, a blunt-tip syringe needle was used for the extrusion process, but inconsistencies in dispensing the viscous solution with the required precision led to the strategic replacement with a tapered needle (Figure 1a_3_). This modification significantly improved performance, especially for applications involving thin-film membranes. The tapered needle’s gradual reduction in diameter enhanced flow dynamics, providing superior control and consistency in printing. This level of precision is critical when dealing with single layers of 100–150 μm thickness, ensuring the successful fabrication of membranes with the desired structural integrity and repeatability.

Additionally, an alternative printing orientation (woodpile arrangement)^57^ was employed for multilayer membranes, where the upper layer was printed perpendicular to the lower layer, as shown in Figure 1a**_3_**. The perpendicular orientation of the printed layers plays a crucial role in enhancing the mechanical strength of the membrane by providing a crossed support structure that resists deformation under pressure. This alternating print direction also minimizes the likelihood of ink fusion between layers during the printing process, ensuring well-defined, distinct boundaries that preserve the intended pore architecture. As a result, the membrane maintains consistent performance, with improved structural integrity and reduced risks of delamination or collapse during filtration operations.

Three distinct solution compositions were used for each layer: 50 wt % PEG for the top layer, 40 wt % PEG for the middle layer, and 30 wt % PEG for the bottom layer (Figure 1a_2_). Varying the PEG concentrations in each layer of the MHM is intended to create a gradient in pore size and porosity, which enhances the overall filtration performance. Higher PEG concentrations in the top layer increase hydrophilicity and create larger pores, facilitating high water flux, while lower concentrations in the bottom layer provide tighter pore structures for effective contaminant rejection. This strategic variation not only optimizes the balance between flux and selectivity but also improves the structural stability of the membrane under operational conditions. After printing the first layer, the process was briefly paused to switch syringes and load the solution for the second layer. This procedure was repeated for the third layer. Figure 1b_1_,c illustrate the oil–water filtration setup and the Flux Recovery Ratio (FRR) of the various membranes tested, respectively. The stirred cell setup in Figure 1b_1_ effectively demonstrates the filtration capabilities of the multilayered hierarchical membrane, showing a marked reduction in oil content after filtration. The MHMs efficiently separate oil droplets from the water phase, significantly lowering the turbidity and oil concentration of the emulsion. The radar chart in Figure 1c illustrates the multilayer membrane’s superior FRR compared to other membrane compositions, demonstrating its exceptional ability to maintain high water flux while effectively rejecting oil contaminants.

### 3.2 Ink Preparation and Rheological Properties

For DIW printing, the ink is better for exhibiting shear thinning behavior, as controlled viscosity is crucial for smooth flow through the nozzle, especially when printing intricate structures.^58,59^ To explore this, inks with varying PVDF concentrations of 10, 15, 20, and 25 wt % in DMAc (designated as 10PVDF, 15PVDF, 20PVDF, and 25PVDF) were prepared. Figure 2a_1_–a_3_ illustrates the procedure of ink preparation, and the printability of the ink for the formation of thin layers was carefully evaluated through the study of rheological behavior depicted in Figure 2b. An increase of 5 wt % in PVDF content significantly impacted the viscosity: at 10 wt %, the viscosity was below 1 Pa·s, which increased to 2–10 Pa·s for 15 and 20 wt %, respectively, and exceeded 10 Pa·s at 25 wt % PVDF. All solutions display a shear-thinning behavior, a desirable property for DIW that facilitates smooth extrusion during printing.

The viscosity of the prepared blends is influenced by the concentration, molecular weight, and structural characteristics of the polymer, along with the additives present in the solvents.^60,61^Figure 2b_2_,b_3_ depicts the variation in viscosity for different percentages of PEG600 and PEG1K. The impact of incorporating PEG600 (20PVDF30PEG600, 20PVDF40PEG600, 20PVDF50PEG600) and PEG1K (20PVDF30PEG1K, 20PVDF40PEG1K, 20PVDF50PEG1K) at different PEG weight percentages (30, 40, and 50) was investigated. Blending with PEG600 resulted in a slight viscosity reduction, with 30 wt % PEG600 the viscosity is lowered to 8.91 Pa·s, which further decreased with higher PEG content. A similar trend was observed with PEG1K, where the viscosity decreased to 8.7 Pa·s at 30 wt % and 7.7 Pa·s at 50 wt % PEG1K. This reduction is attributed to the low molecular weight of PEG, which helps to create homogeneous solutions when mixed with PVDF, thereby reducing viscosity.^62^ Additionally, PEG1K demonstrated better control over viscosity adjustments across different weight percentages. These findings are consistent with previous studies^63^ where the viscosity variation between low and slightly higher molecular weight PEG (600 vs 1K) is linked to an increase in macromolecule size and chain length. The extended chains of PEG1K enhance intermolecular entanglement within the blend, contributing to the observed changes in viscosity.

In addition, this behavior could be linked to the solubility parameters of the solvent and polymer. Specifically, the overall solubility parameter (δ) is divided into three individual components, namely, dispersive force (δ_d_), dipole interaction (δ_p_), and hydrogen bonding (δ_h_), combining into an overall solubility parameter as discussed in ref. (64) given by
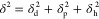
4

Using Hoy’s method, the overall solubility parameter for DMAc is calculated to be 22.1 (J/cm^3^)^1/2^.^65^ Similarly, studies have reported the solubility parameters of PEG600 and PEG1K as 19.7 and 21.3 (J/cm^3^)^1/2^, respectively.^66^ The observed reduction in viscosity when blending PVDF with different PEGs can be partly attributed to the relatively weaker interaction between PEG600 and DMAc. These values indicate varying degrees of compatibility between the PEG additives and DMAc, affecting how well the polymers interact within the solution. Specifically, the weaker interaction between PEG600 and DMAc contributes to a reduction in the overall viscosity of the PVDF–PEG600 blends, as seen in Figure 2b_2_. As PEG is added, the PEG chains tend to coil more tightly due to poor solubility, which reduces their interaction with the surrounding medium. This contraction minimizes entanglements between PEG and PVDF chains, leading to a notable decrease in viscosity, particularly at higher PEG contents.^63^ This effect is evident in the rheological characterization graphs Figure 2b_2_,b_3_, where increasing PEG content consistently lowers viscosity across varying shear rates, demonstrating enhanced flow properties. Moreover, the differences between PEG600 and PEG1K blends are apparent; PEG1K, with a solubility parameter closer to that of DMAc, exhibits slightly better interactions and control over viscosity variations. As shown in Figure 2b_3_, PEG1K blends maintain a more stable viscosity profile compared to PEG600 blends. This alignment of solubility parameters results in a more homogeneous solution with less internal resistance, highlighting the importance of solvent–polymer interactions in optimizing the ink flow behavior.

### 3.3 Analysis of PVDF–PEG Membrane Properties

#### 3.3.1 Structural and Morphological Studies of Printed Membranes

**Figure 3:**
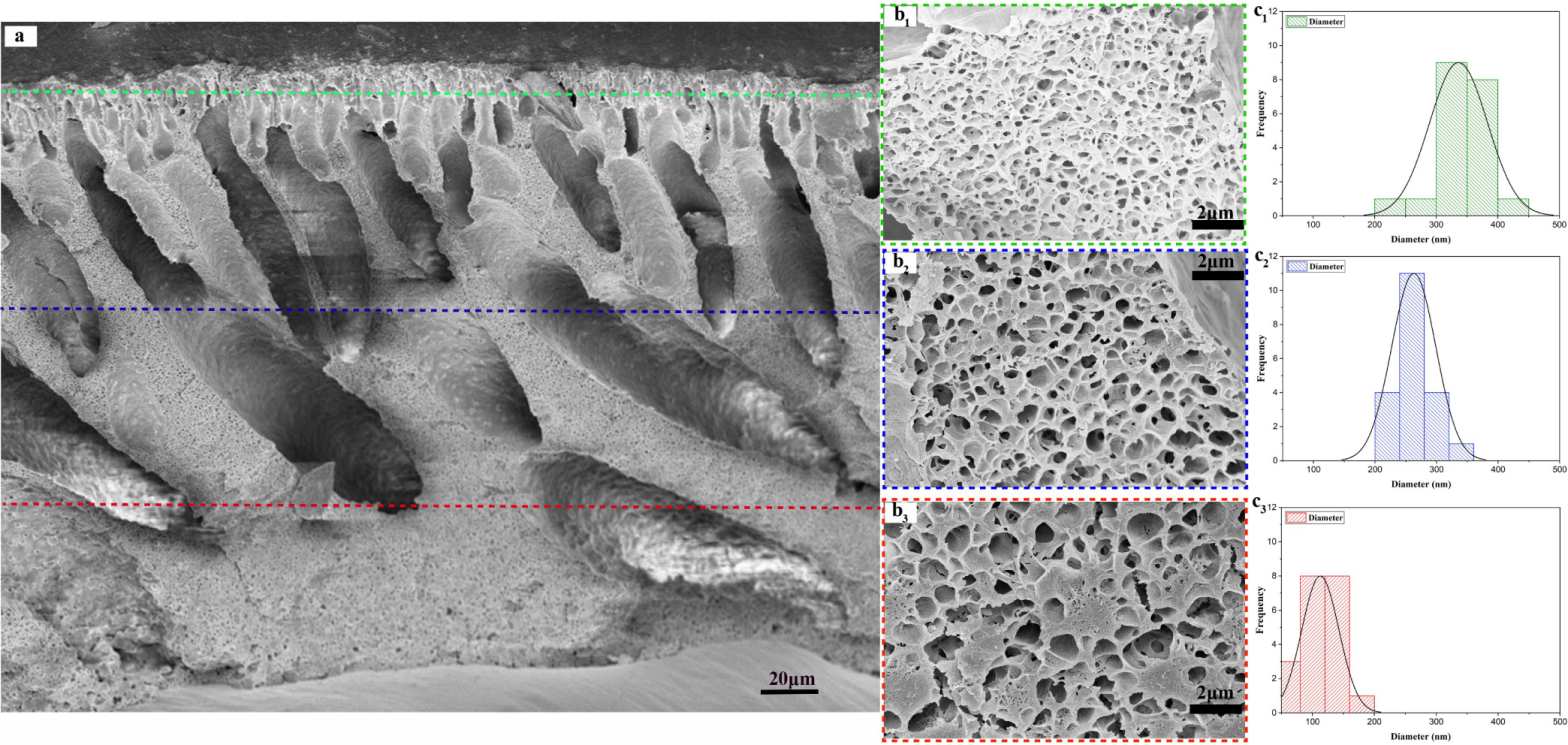
(a) SEM image of the 3D-printed multilayered membrane illustrating the formation of finger-like pores across the layers. (b_1_–b_3_) SEM images showing the cross-sectional pores in the top (20PVDF50PEG1K), middle (20PVDF40PEG1K), and bottom (20PVDF30PEG1K) (see compositions in Table 1) layers of the membrane, respectively. (c_1_–c_3_) Graphs depicting the pore size distribution of the top, middle, and bottom layers, highlight the variation in pore diameters across the different layers of the membrane.

The surface pores and cross sections of the printed single-layered and multilayered membranes were analyzed using SEM and BET techniques. The cross-sectional SEM images of the multilayered membranes (Figure 3a) reveal an asymmetric skin layer at the top, supported by a three-layered porous structure. The top layer is characterized by a dense, thick finger-like structure with interconnected pores, the middle layer features a porous microvoid architecture, and the bottom layer transitions into a sponge-like structure with suppressed finger-like pores. These distinct structural features result from the varying percentages of PEG used during membrane fabrication. The diverse pore architectures are formed during the phase inversion process, where rapid PVDF crystallization occurs upon immersion in the coagulation bath. This crystallization is driven by the diffusion of PEG and DMAc into the nonsolvent (water bath) and the repulsion between the hydrophobic PVDF phase and the hydrophilic PEG phase, leading to the formation of the observed porous structures.^67^

The pore size and distribution at each layer interface are significantly influenced by the PEG concentration, as shown in Figure 3b,c. Specifically, the bottom layer containing 30 wt % PEG1K exhibits an average pore size of 114 nm, which progressively increases to 348 nm in the top layer with 50 wt % PEG1K, as detailed in Table 2. This increase in pore size can be attributed to two main factors: (i) higher PEG content leads to larger volume voids, facilitating the formation of larger pores, and (ii) the phase inversion process progresses from the bottom up, involving complex interactions between the polymer, solvent, and nonsolvent.^68^ Similarly, the single-layered membrane, depicted in Figure S2a–c, demonstrates comparable structural characteristics, with average macropore sizes of 114, 188, and 298 nm corresponding to 30, 40, and 50 wt % PEG1K, respectively, at the cross-sectional interface. The slight variation in pore size between single-layered and multilayered membranes depicted in Figure S3 is due to the increased thickness of the MHM, which enhances nonsolvent diffusion into additional layers. Since DMAc is a more effective solvent for PVDF than PEG, increasing PEG content induces greater instability in the solution, leading to faster phase separation, larger pore formation, and a thicker overall membrane structure.^67,69^

**Table 2:** Surface Morphology Characteristics of the Different Printed Membranes Were Measured by SEM, AFM, and BET Analyses

	SEM	AFM	BET	
Sample	Pore size (nm)	Roughness (nm)	Surface area (m^2^/g)	Pore size (nm)
20PVDF	100 ± 23	26.86	6.84	NA
20PVDF30PEG1K	114 ± 31	21.19	8.81	16.83
20PVDF40PEG1K	188 ± 62	23.74	9.13	16.13
20PVDF50PEG1K	298 ± 60	23.17	9.45	17.36
Multilayered	114 ± 34 (bottom)	23.97	12.05	20.82
	262 ± 36 (middle)			
	348 ± 66 (top)			

Surface analysis of single-layered and multilayered membranes (Figures S2 and S3) demonstrates a nanoscale surface area increase in higher PEG content in the blend, consistent with the BET analysis results presented in Table 2. BET analysis showed that all single-layered 3D-printed membranes formed from PVDF–PEG1K blends exhibited an increased surface area compared to the neat PVDF membrane. Notably, the multilayered membranes displayed a significantly higher surface area from the BET measurements, reaching up to 12.05 m^2^/g. This enhancement directly influences filtration efficiency, marking a significant step forward in optimizing the performance characteristics of the membranes.^70,71^

The membrane surface roughness plays a crucial role in determining adhesion and fouling behavior. Typically, reduced surface roughness can slightly alter the effective filtration area while enhancing antifouling properties.^72^ AFM analysis, conducted at a scan size of 5 μm, was used to evaluate the roughness (RMS) of the membranes (Table 2). For PVDF–PEG blends, a slight reduction in surface roughness was noted. Although the addition of PEG increased the overall porosity, it did not significantly impact the surface roughness, thereby maintaining high selectivity and enhancing the surface area. This characteristic minimizes the potential leaching of filtered materials into the permeate and imparts superior antifouling properties to the printed membranes.

#### 3.3.2 Chemical Composition Influences on Physical Structures

The surface chemical composition of the PVDF and Multilayer membrane was studied by XPS measurements. Peaks of C 1s can be clearly identified in both spectra (Figure S4a) indicating the existence of PVDF on the surface. As shown in Figures S4b,c for pure PVDF and the Multilayer respectively, the peaks of C 1s can be deconvoluted into 4 peaks C–C, CH_2_, C–OH, and CF_2_ which are reflective of groups directly bonded to the carbon. The characteristic peaks of PVDF exist in both spectra, peaks at 282.1, 283.7, 285.7, and 288.1 eV are attributed to (C–C), (CH_2_), (C–OH), and (CF_2_) respectively. The absence of any shift in the binding energies of the peaks indicates that no significant chemical reaction occurred between PVDF and PEG. The decreased intensity of the CF_2_ peak in the multilayer suggests a decrease in the fluorine-related surface functional groups, consistent with the physical bonding between the PVDF and PEG.^73^

The FTIR spectra of neat PEG, PVDF, and blends of PVDF–PEG were compared to follow the chemical bonding between the PVDF and PEG and the blending effect of different wt % of PEG on the printed membrane. Certainly, the porogen PEG is mainly dissolved in water during the phase inversion process as proven by the FTIR spectra (Figure S5). Accordingly, during solution preparation, the PEG and PVDF are physically attached through weak hydrogen bonding, dipole–dipole, and Van Der Waals interactions that can be easily broken when the membrane is immersed in the coagulation bath. However, the effect of this blend is prominent on the PVDF as shown in spectra Figure S6a for PVDF–PEG1K blends and Figure S6b for PVDF–PEG600 blends. All the absorption peaks shown in the graph are characteristic of PVDF and originated from -C = C stretching, bending, and −C-H bending vibrations. In particular, peaks around 1170 cm^–1^ refer to −CH_2_ rocking vibrations, and peaks around 875 cm^–1^ represent −CF_2_ asymmetric stretching vibrations.^74^ According to the FTIR spectra, the dipole–dipole interactions are possible, verified by the slight decrease in absorbance band intensity in the PVDF–PEG blends. Moreover, the FTIR data showed peaks of alpha phase PVDF crystal at 761, 795, and 975 cm^–1^, and a peak at 839 cm^–1^ assigned to the beta phase crystal of PVDF.^75^ The intensity of α phase peaks slightly decreases with the increase of PEG in the blend, and the fraction in the blend can be calculated using the information below.^76^
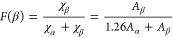
5where *X*_α_ and *X*_β_ are the mass fraction of α and β crystalline phases, and *K*_α_, *K*_β_ are the absorption coefficients at a particular wavelength. *K*_α_ is 6.1 × 10^4^ cm^2^/mol, and *K*_β_ is 7.7 × 10^4^ cm^2^/ mol. *A*_α_, *A*_β_ are the area of absorption bands at 761 and 839 cm^–1^ respectively. The results indicate an increase in the beta fraction with the increase of PEG wt % in the blend compared to neat PVDF which probably happens during the preparation/printing procedures, where PEG possesses enough hydroxyl group (−OH) to nucleate the PVDF chains into β-phase.^77,78^

**Figure 4:**
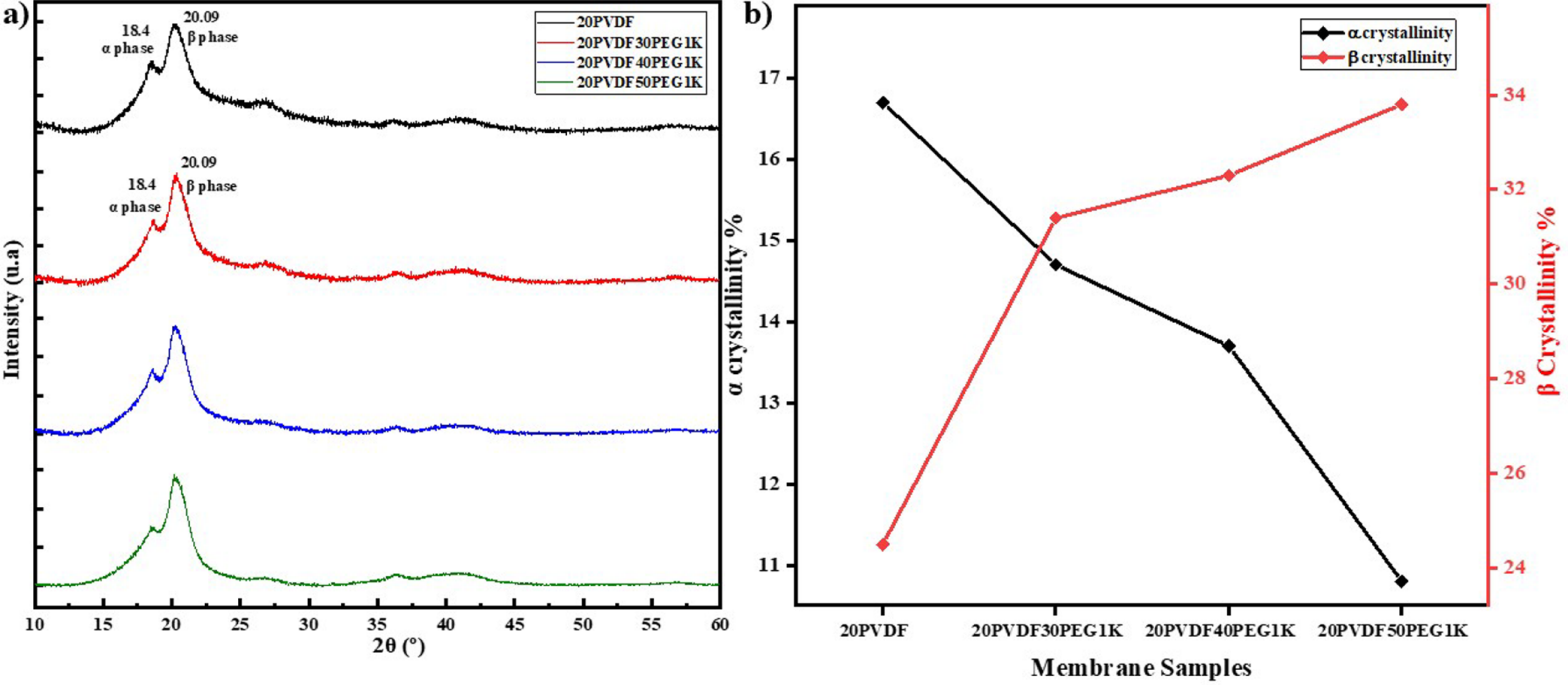
(a) XRD diffractograms of various PVDF–PEG 1K blends, showcasing the crystalline structure of the different formulations. (b) Evolution of the α (alpha) and β (beta) crystalline phases of PVDF across the different membranes, highlighting the changes in phase composition with varying PEG content.

To better understand the impact of PEG on the crystallization behavior of PVDF, XRD measurements were conducted. Figure 4a displays the XRD diffractograms of various PVDF–PEG1K blends, highlighting the crystalline structure of the different formulations, while Figure S7 shows the diffractograms for PVDF–PEG600 blends. All samples exhibit the semicrystalline nature of PVDF, marked by distinct peaks at 2θ = 18.4° and 20.09°, corresponding to the α-phase and β-phase crystalline structures of PVDF, respectively.^79^ As the PEG content increases, a noticeable reduction in the area of the α-phase peaks is observed, accompanied by a slight broadening of the β-phase peak. Crystallinity calculations, shown in Table 3, indicate that the α-phase crystallinity decreases from 14.7% to 10.8% as the PEG1K content rises from 30 wt % to 50 wt %, while the β-phase crystallinity increases from 31.4% to 33.8%. This shift confirms the role of PEG in disrupting PVDF chain packing, reducing chain entanglement, and promoting phase transformation from the α-phase to the β-phase. Interestingly, the overall crystallinity (*X*_c_) calculated via eq 5 remains relatively stable, decreasing only slightly from 49.3% to 47.8%, suggesting that the changes in α- and β-phase crystallinity are primarily due to phase transformations rather than a reduction in overall crystallinity caused by PEG interactions during the membrane preparation and printing processes.^75,79,80^
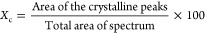
6

**Table 3:** Degree of Crystallinity for the α, and β Phases and Overall Crystallinity (*X*_c_) for the Different Membranes

Composition	*X*_c_ α-phase	*X*_c_ β-phase	*X*_c_ (%)
20PVDF	16.7	24.5	49.3
PEG600 samples			
20PVDF30PEG600	15.5	26.2	45.9
20PVDF40PEG600	13.2	32.6	44.6
20PVDF50PEG600	9.8	31.1	46.9
PEG1K samples			
20PVDF30PEG1K	14.7	31.4	49.1
20PVDF40PEG1K	13.5	32.3	49.1
20PVDF50PEG1K	10.8	33.8	47.8

In addition to PEG1K, the influence of PEG600 on PVDF crystallinity is also notable, as seen in Figure S7. Unlike PEG1K, which primarily shifts PVDF crystallinity toward the β-phase, PEG600 leads to a more pronounced reduction in the α-phase crystallinity, from 16.7% in pure PVDF to 9.8% in the 20PVDF50PEG600 blend. This significant drop in α-phase crystallinity suggests that PEG600 disrupts the crystalline lattice more effectively, reducing the orderly packing of PVDF chains. Moreover, Table 3 indicates that PEG600 also enhances the β-phase crystallinity, reaching 31.1% at 50 wt % PEG600, but to a slightly lesser extent compared to PEG1K blends. This subtle difference could be attributed to the lower molecular weight and smaller chain size of PEG600, which might lead to less pronounced interactions with PVDF but still contribute to β-phase formation by facilitating greater chain mobility.

Figure 4b further illustrates the evolution of the α and β crystalline phases across the different membranes, showing how varying PEG1K content directly influences the phase composition of PVDF. In contrast, the overall crystallinity (*X*_c_) of the PVDF–PEG600 blends shows a distinctive trend compared to PEG1K blends, as detailed in Table 3. For the PEG600 blends, the *X*_c_ decreases more sharply from 49.3% in pure PVDF to 45.9% and 44.6% at 30 and 40 wt % PEG600, respectively, before slightly rising to 46.9% at 50 wt %. This pattern suggests a more dynamic rearrangement of crystalline domains in the presence of PEG600, potentially due to faster phase separation during membrane fabrication. Such changes in crystallinity can impact the mechanical properties of the membranes, as the reduced overall crystallinity can lead to softer membranes that may offer enhanced flexibility but might compromise mechanical strength under high-pressure conditions. The modifications in crystallinity driven by PEG600 and PEG1K have direct implications on the membrane performance. The reduction in α-phase crystallinity and a corresponding increase in β-phase crystallinity enhance the polar properties of the membranes, which can improve hydrophilicity and water flux. Additionally, the lower crystallinity observed with higher PEG600 content may contribute to better fouling resistance, as the more amorphous regions allow for easier cleaning and reduced particle adhesion. However, this could come at the cost of reduced tensile strength, indicating a trade-off between enhancing membrane permeability and maintaining robust mechanical properties.

Besides, the observed decrease in the α-phase and corresponding increase in the β-phase align with rheological data, particularly viscosity measurements, supporting the conclusion that increased PEG content reduces intermolecular forces in PVDF, enhances polymer chain mobility, and contributes to the distinct phase behavior observed in the XRD analysis.

#### 3.3.3 Mechanical Properties of Membrane

The mechanical properties of the membranes, including Young’s modulus, elongation, tensile strength, and stiffness, are summarized in Table 4, while the stress–strain curves for each 3D-printed membrane are shown in Figure S8. The PVDF–PEG membranes exhibit lower tensile strength and elongation compared to pure PVDF membranes, indicating increased brittleness in PVDF–PEG composites. This increase in brittleness can be attributed to the expanded pore distribution caused by the addition of PEG, as confirmed by SEM and BET analyses. Interestingly, the membranes prepared with PEG600 show improved elongation-at-break and stiffness compared to those prepared with PEG1K. For example, the 20PVDF30PEG600 blend exhibits a stiffness of 4.62 N/mm and an elongation of 3.62%, compared to 2.36 N/mm stiffness and 2.48% elongation for 20PVDF30PEG1K. This behavior is likely due to the slightly lower degree of crystallinity in PEG600 blends, resulting in a more deformable matrix that enhances flexibility,^72,80^ whereas the higher crystallinity of PEG1K blends restricts elongation due to longer, less deformable polymer chains.

**Table 4:** Mechanical Properties of the Different Membranes in Dry States

Composition	Stiffness (N/mm)	Young’s Modulus (MPa)	Elongation (%)	Tensile Strength (MPa)
15PVDF	2.80 ± 0.30	53.90 ± 8.43	1.35 ± 0.42	2.18 ± 0.13
20PVDF	4.59 ± 0.52	114.80 ± 0.55	6.81 ± 0.50	2.78 ± 0.72
PEG600 samples				
20PVDF30PEG600	4.62 ± 0.19	96.30 ± 1.09	3.62 ± 0.81	2.45 ± 0.35
20PVDF40PEG600	2.69 ± 0.35	56.10 ± 0.35	3.63 ± 0.44	2.10 ± 0.17
20PVDF50PEG600	3.20 ± 0.26	66.70 ± 0.26	3.21 ± 0.61	2.49 ± 0.23
PEG1K samples				
20PVDF30PEG1K	2.36 ± 0.44	49.20 ± 0.45	2.48 ± 0.47	2.78 ± 0.13
20PVDF40PEG1K	3.26 ± 0.39	68.10 ± 0.71	2.90 ± 0.55	1.94 ± 0.09
20PVDF50PEG1K	2.28 ± 0.09	47.60 ± 0.13	2.26 ± 0.45	2.11 ± 0.29
Multilayered samples				
Multilayer	0.73 ± 0.08	14.04 ± 0.26	0.70 ± 0.04	2.87 ± 0.36

The multilayer membrane stands out with the highest tensile strength among all samples, reaching 2.87 MPa, despite having the lowest Young’s modulus (14.04 MPa) and stiffness (0.73 N/mm). The phase separation occurring during the phase inversion process leads to the formation of distinct domains: regions rich in PEG and regions rich in PVDF. The PVDF-rich phase enhances resistance to deformation and contributes significantly to the overall strength of the membrane due to the different semicrystalline PVDF-rich layers. This superior tensile strength is attributed to its unique structural composition and significantly increased thickness of approximately 250 μm.^81^ The combination of dense top layers, microvoids in the middle, and sponge-like structures at the bottom, observed through SEM imaging, contributes to the enhanced mechanical strength of the multilayer membrane, allowing it to distribute and absorb applied stress effectively.^82^ The gradual increase in PVDF concentration, particularly in 15PVDF and 20PVDF membranes, further supports the enhancement of mechanical properties, as seen with a rise in tensile strength from 2.18 to 2.78 MPa, respectively. This trend underscores the role of PVDF as a reinforcing component within the membrane matrix. Additionally, the presence of interconnected finger-like and sponge-like pores in the multilayer structure helps maintain a balance between strength and flexibility, making these membranes highly suitable for demanding applications such as wastewater treatment, where resistance to mechanical stress is crucial.^62,83^ The membrane was subjected to filtration for 3 continuous cycles, each lasting for more than 100 min at a pressure of 1 bar, and no cracking or defects were noticed, the image depicted in Figure S9 shows that the membrane is robust and can effectively handle similar pressure applications without compromising its structural integrity. The fabrication involving the use of MHMs with the advantage of interchanging different layer materials has improved its ability to enhance reliability for new applications. The mechanical robustness demonstrated by these multilayered membranes positions them as ideal candidates for high-performance filtration applications.

### 3.4 Membrane Performance

**Figure 5:**
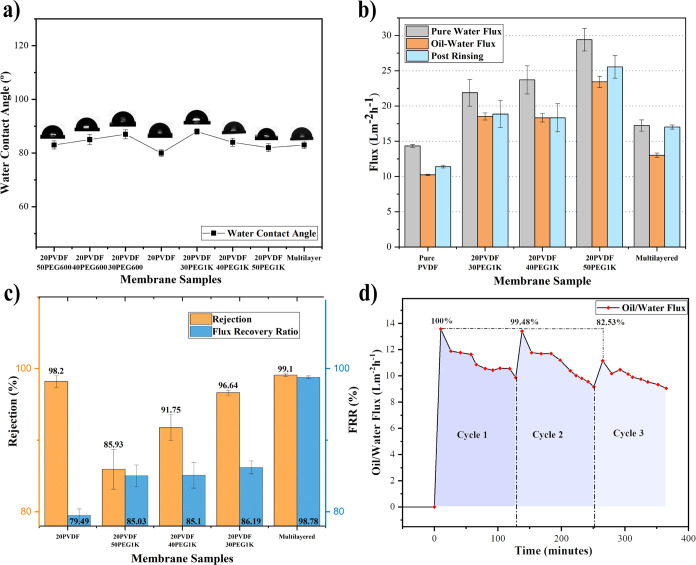
(a) Water contact angle (WCA) measurements of various 3D-printed membranes, indicating surface wettability differences among the membrane samples. (b) Performance assessment of the membranes, showing pure water flux, oil–water flux, and pure water flux after rinsing, demonstrating the impact of fouling on flux recovery. (c) Comparison of flux recovery ratio and rejection efficiency of the different membranes, highlighting their separation performance. (d) Analysis of the fouling behavior and reusability of the multilayer membrane over multiple cycles, illustrating its stability and effectiveness in maintaining oil–water flux over time.

Membrane performance was evaluated through water contact angle (WCA) measurements and flux testing under various conditions. Figure 5a shows the WCA results for the different membranes, indicating that the incorporation of PEG, despite forming pores and slightly reducing surface roughness as observed in AFM analysis, did not significantly impact the membranes’ wettability. All tested membranes, including those made from PVDF–PEG blends, exhibited consistent hydrophilic behavior with contact angles around 80°, demonstrating their suitability for oil–water separation applications. Additionally, all printed membranes exhibit an underwater oleophobic character as shown in Figure S10a. All the prepared membranes have an underwater contact angle (UWCA) around 110° that increases with the MHMs underscoring a UWCA of 155° explaining the higher oil rejection. The MHMs were meticulously printed to conform to the precise geometry of the holder, ensuring optimal integration for subsequent performance characterization within a stirred cell to facilitate a comprehensive evaluation of both pure water filtration and oil–water separation efficiencies. Pure water flux performance was assessed following a 3-h compaction process using DI water, as shown in Figure 5b. This step was essential to stabilize the membranes, reduce initial flux decline, and improve mass transfer across the membrane surface, ultimately enhancing their performance during subsequent filtration. The tested membranes included pure PVDF and blends with 30, 40, and 50 wt % PEG600 and PEG1K, revealing how different PEG concentrations influence flux and fouling behavior. The compaction step and flux measurements were conducted in a 200 mL stirred cell filled with DI water. The results highlight the effectiveness of the membranes in maintaining stable flux rates, particularly for oil–water separation, and emphasize the importance of the precompaction process in optimizing membrane performance during filtration.

The results in Figure 5b highlight two important findings: First, all PVDF–PEG blend membranes demonstrated enhanced pure water flux compared to the pure PVDF membranes. Second, a direct correlation was observed between pure water flux and PEG content, emphasizing the significant influence of PEG on membrane performance. This improvement is primarily attributed to the increased membrane pore size and porosity, as confirmed by SEM and BET analyses. Notably, the 20PVDF50PEG1K membrane exhibited the highest pure water flux, reaching 29.4 L·m^–2^·h^–1^, which represents a 2-fold improvement over the pure PVDF membrane, which had a flux of 14.34 L·m^–2^·h^–1^. Additionally, the molecular weight of PEG plays a crucial role in membrane performance. As shown in Figure S10b, membranes incorporating higher molecular weight PEG, such as PEG1K, outperformed those with lower molecular weight PEG600, with 20PVDF50PEG600 achieving a flux of 27.35 L·m^–2^·h^–1^. This performance enhancement is linked to the increased accumulation and interaction of PEG within the membrane matrix as its molecular weight rises, which in turn leads to an expansion of pore size and improved water flux.

The rejection performance of the printed membranes was evaluated using synthetic oil–water emulsions. During the filtration process, the magnetic stirrer was set to 200 rpm to minimize concentration polarization and reduce early fouling effects.^84^ As shown in Figure 5b, the oil–water flux was lower than the pure water flux due to the retention of oil particles. However, all PVDF–PEG blend membranes exhibited higher oil–water fluxes compared to pure PVDF, with the highest flux recorded at 23.43 L·m^–2^·h^–1^ for 20PVDF50PEG1K, compared to 10.23 L·m^–2^·h^–1^ for pure PVDF. The flux recovery ratio (FRR) and rejection rate results, shown in Figure 5c, indicated that all blend membranes achieved higher FRR values than pure PVDF (79.49%). Notably, membranes prepared with PEG1K demonstrated more consistent flux recovery, achieving FRR values of 86.19%, 85.1%, and 85.03% for 30, 40, and 50 wt % PEG1K, respectively. In comparison, membranes with PEG600 showed varying FRR values of 88.46%, 82.83%, and 76.19% for 30, 40, and 50 wt % PEG600, respectively, as shown in Figure S10c. Additionally, PEG1K-based blends consistently achieved higher rejection rates compared to those with PEG600, highlighting the PEG1K’s superior performance in enhancing the membrane’s separation efficiency and recovery properties.

Based on these findings, a multilayered membrane configuration using PEG1K was developed, with layers of 30, 40, and 50 wt % PEG1K forms the base, middle, and top layers, respectively. This configuration was chosen for its outstanding performance in pure water flux, oil–water rejection, and flux recovery ratio. By strategically layering these membranes, the design aims to maximize their individual strengths to create a highly efficient filtration system. The MHM, characterized by a cross-sectional SEM showing a hierarchical porous structure and a thickness of 250 ± 5 μm, achieved a pure water flux of 17.23 L·m^–2^·h^–1^ and an oil–water flux of 13.01 L·m^–2^·h^–1^ showing high precision and reproducibility of the process, reflecting consistent performance and minimal variability across measurements. The concept of fabricating MHMs with varying pore sizes, inspired by soil structures, has demonstrated remarkable success in enhancing the performance and reproducibility of filtration membranes. This approach focuses on developing cost-effective membranes using a straightforward preparation method, ensuring ease of reproducibility. The incorporation of porogens not only improved water flux but also simultaneously enhanced the FRR and rejection efficiency, a significant advancement for MHMs. Comparative analyses, as summarized in Figure S11, reveal that achieving high rejection rates without compromising the FRR remains a formidable challenge across most studies. In contrast, the MHM developed in this study exhibited exceptional performance, achieving the highest FRR of 98.7% and a superior rejection rate of 99.02%, surpassing single-layer membranes and other blends, including pure PVDF. Furthermore, the MHM retained a consistently impressive flux rate of 99.48% after 120 min of continuous filtration, demonstrating its robustness and reusability in oil–water filtration applications. Its stability and reusability were further highlighted by a minimal decline in flux from 14 to 9.05 L·m^–2^·h^–1^ after over 400 min of continuous oil–water emulsion filtration, as shown in Figure 5d, showcasing its excellent fouling resistance compared to single-layered membranes.

## 4 Conclusion

This study successfully demonstrated the fabrication of MHMs using DIW 3D printing technology, marking a significant advancement in additive manufacturing for water treatment applications. Single-layer membranes composed of various blends of PVDF and PEG were systematically analyzed, leading to the development of MHMs specifically designed for oil–water emulsion treatment. Utilizing PVDF and PEG1K blends with 50, 40, and 30 wt % from top to bottom, the multilayer membranes were printed with a sequential alternating printing direction to prevent layer fusion, achieving a final thickness of 250 μm. The as-fabricated MHMs exhibited outstanding performance in water filtration, achieving a flux recovery ratio of 98.7% and the highest rejection rate of 99.02%, outperforming traditional single-layer membranes. This innovative approach showcases the potential of combining 3D printing technology with tailored membrane compositions to create efficient, customizable filtration systems. The success of these MHMs underscores the potential of DIW 3D printing in advancing membrane technology, opening new avenues for the development of highly specialized membranes for water treatment and other separation processes.

## Supporting Information Available

G-code visualization of 3D printed membranes, SEM images, XPS spectra, XRD and FTIR, stress–strain curves, image of MHM, underwater contact angle, membrane performance graphs, and comparison of literature studies (PDF)
