# Supplementary material for: Biomimetic 3D Prototyping of Hierarchically Porous Multilayered Membranes for Enhanced Oil–Water Filtration
Source: ACS Appl Mater Interfaces. 2025 Jan 23;17(5):8285–98. doi: 10.1021/acsami.4c18528 (PMC11803563; doi:10.1021/acsami.4c18528)
Supplement: Supplementary file 1 — am4c18528_si_001.pdf [file am4c18528_si_001.pdf]

Supporting Information

Biomimetic 3D Prototyping of Hierarchically Porous Multilayered Membranes for Enhanced Oil-Water Filtration

Abhishek Saji Kumar[a], Rayane Akoumeh[b], Arunachalam Ramanathan[c], JaeWoo Park[a], Varunkumar Thippanna[c], Dhanush Patil[c], Yuxiang Zhu[d], Dharneedar Ravichandran[e], Sri Vaishnavi Thummalapalli[c], M. Taylor Sobczak[c], Lindsay Bick Chambers[c], Taylor G. Theobald[c], Churan Yu[c], Chao Sui[c], Libin Yang[c], Deepalekshmi Ponnamma[b], Mohammad K. Hassan[b*], Maryam Al-Ejji[b*], Sui Yang[af*], Kenan Song[g, h*]

[a] *Materials Science and Engineering, School for Engineering of Matter, Transport and Energy (SEMTE), Ira A. Fulton Schools of Engineering, Arizona State University (ASU), Tempe, AZ, USA, 85281*
[b] *Center for Advanced Materials, Qatar University, P.O. BOX 2713, Doha, Qatar*
[c] *Mechanical Engineering, College of Engineering, University of Georgia, 302 E. Campus Rd, Athens, GA 30602*
[d] *School of Manufacturing Systems and Networks (MSN), Ira A. Fulton Schools of Engineering, Arizona State University (ASU), Mesa, AZ, USA, 85212*
[e] *Department of Mechanical Engineering, University of California, Berkeley, CA, USA 94720*
[f] *Center for Molecular Design and Biomimetics at the Biodesign Institute, Arizona State University, Tempe, AZ, USA, 85281*
[g] *Associate Professor of Mechanical Engineering, College of Engineering, University of Georgia (UGA), 302 E. Campus Rd., Athens, USA, 30602*
[h] *Adjunct professor at the School of Manufacturing Systems and Networks (MSN), Ira A. Fulton Schools of Engineering, Arizona State University (ASU), Mesa, AZ, USA, 85212*

[*]Corresponding author, Email: kenan.song@uga.edu, sui.yang@asu.edu, mohamed.hassan@qu.edu.qa, maryam.alejji@qu.edu.qa

Table of Contents

1. **Various AM membrane preparation techniques: 5**
2. **Printing Technique 6**
3. **Membrane Microstructures 8**
4. **Membrane Properties 14**

Figure S1. (a) shows G-code for printing the MHMs with alternating layer-by-layer printing. (b) shows the layer thickness compared to 1 cent coin with a thickness of 1.52 mm. 7
Figure S2. Cross-sectional pore morphology of single-layer membranes with varying PEG1K content: (a) 30 wt.%, (b) 40 wt.%, and (c) 50 wt.%. Corresponding pore size distributions are shown in (a_1), (b_1), and (c_1), illustrating an increase in pore size with higher PEG1K content. 8
Figure S3. This figure shows the surface pores of the membranes, (a)-(e) denoting the surface pores of 20PVDF30PEG1K, 20PVDF40PEG1K, 20PVDF50PEG1K, 20PVDF, and the Multilayered membrane, respectively. 9
Figure S4. (a) XPS spectra of PVDF and Multilayer membranes, (b), (c) C 1s spectra of PVDF and Multilayer respectively. 10
Figure S5. FTIR Absorbance spectra of PEG1K, PVDF, and PVDFPEG1K. 11
Figure S6. FTIR absorbance spectra with (a): PVDF and PVDF-PEG1K blends and (b) PVDF and PVDF-PEG600 blends spectra. 12
Figure S7. XRD diffractogram of different PVDF-PEG 600 blends. 13
Figure S8. (a)-(h) Stress-strain curves illustrating the mechanical behavior of the various 3D-printed membranes, providing insights into mechanical characteristics under applied stress. 14
Figure S9. Image of MHM on the stirrer cell after 3 cycles of continuous filtration. 15
Figure S10. Characteristics and performance metrics of PVDF-PEG600 blend membranes: (a) Underwater contact Angle of different printed membranes. (b) Pure water flux before and after oil-water filtration, demonstrating the membrane's durability and filtration capability; (c) Oil rejection percentage from the oil-water solution and membrane reusability, indicated by the flux recovery ratio, highlighting consistent performance across multiple filtration cycles. 16
Figure S11. The graph shows the comparison of flux recovery ratio (FRR %) and rejection % from polymer membranes prepared using different techniques highlighting the superior performance of MHMs. 17

Table S1. The current state-of-the-art of the 3D printable membranes 5

1. Various AM membrane preparation techniques:

Additive manufacturing and 3D printing methodologies are widely applied across diverse industrial sectors, streamlining and expediting synthesis processes. Among the prevalent techniques employed in membrane fabrication through additive manufacturing, notable methodologies include Fused Deposition Modelling (FDM), Selective Laser Sintering (SLS), Stereolithography (SLA), Direct Ink Writing (DIW), Digital Light Processing (DLP), and others. However, it is imperative to note that these additive manufacturing techniques are accompanied by inherent drawbacks such as material limitations, suboptimal mechanical properties, the necessity for support structures, elevated costs, significant waste generation, challenges in achieving high surface finish, and diminished accuracy.[1] The FDM printer has been introduced in the membrane field for membrane preparation and feed spacer preparation.[2] **Table S1** presents a comprehensive overview of the printing techniques and their comparative analysis.

Table S1. The current state-of-the-art of the 3D printable membranes

3D printing Method	Materials	Pore size	Thickness (µm)	Strength (MPa)	Applications	References
**SLA**	Slurry mixture*	14 nm	230	-	-	[3]
**FDM**	Nafion	-	200†	-	Ion exchange	[4]
**SLS**	Polyamide-12	14.5 µm	600	4.2-28	microfiltration	[5]
**DIW**	PVDF/PEG	7-20 nm	150	2.8	Oil-water rejection	This Work

† First layer thickness.

*Mixture of Solvent, photopolymer, ceramic and dispersant

2. Printing Technique

In our study, we tackled the challenge of achieving high repeatability in printing membranes with a thickness of 150 microns amidst variations in fluid viscosity. While mechanical and fluid dispenser-assisted printing methods produced membranes with desirable thicknesses, the fluid dispenser emerged as the most viable option. Despite its perceived complexity, the liquid dispenser offers greater control and adaptability, as adjustments in pressure alone can yield commendable results when using diverse composition solutions. In our investigation, we grappled with adjusting parameters such as the pulse/μl or the material flow rate multiplier in the printing software for each new solution to ensure consistent membrane thickness. This iterative process demands considerable time and attention during printing to calibrate or achieve optimal flow conditions. These findings underscore the crucial role of parameter optimization in maintaining uniformity in membrane fabrication, highlighting the need for streamlined protocols or automated systems to enhance efficiency and reproducibility in such intricate printing processes.

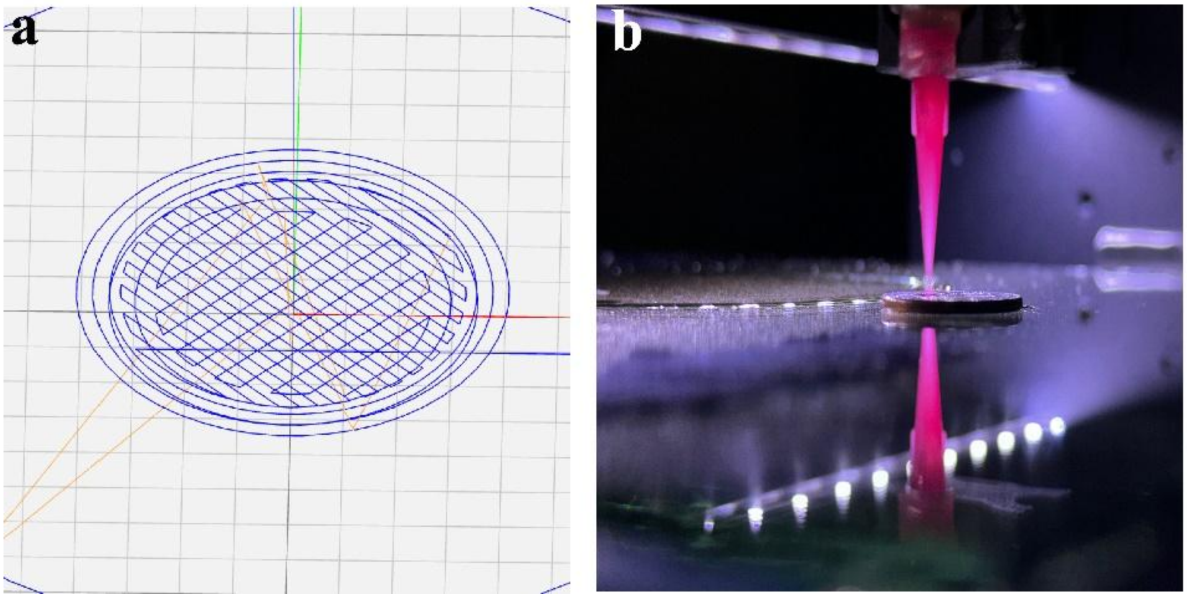

Figure S1. (a) shows G-code for printing the MHMs with alternating layer-by-layer printing. (b) shows the layer thickness compared to 1 cent coin with a thickness of 1.52 mm.

3. Membrane Microstructures

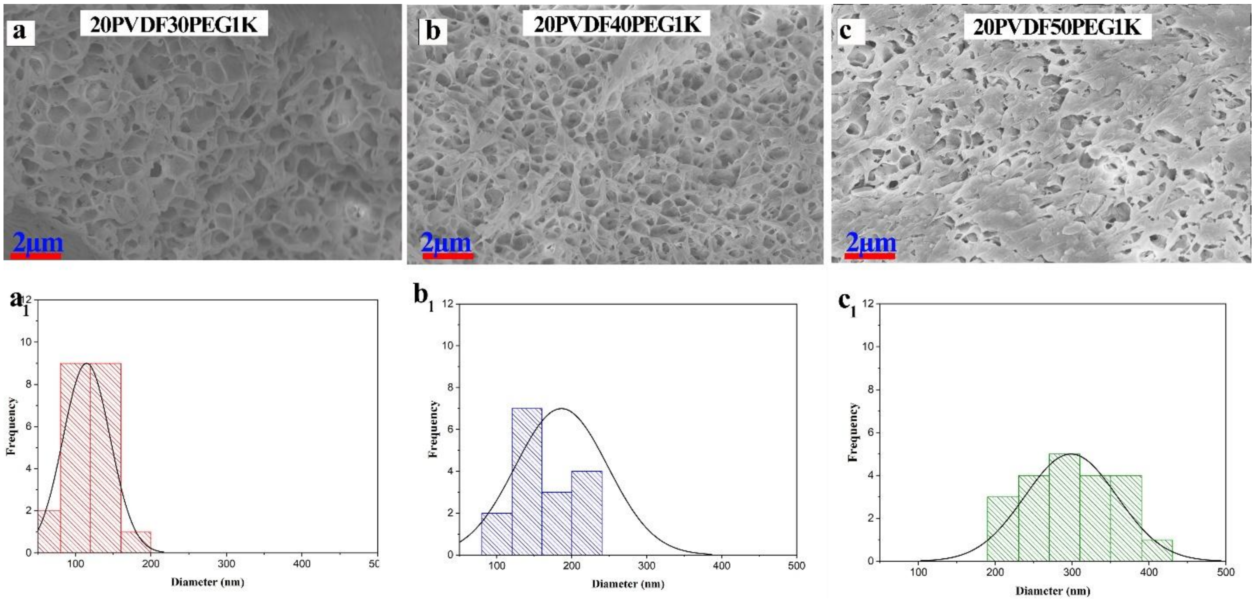

Figure S2. Cross-sectional pore morphology of single-layer membranes with varying PEG1K content: (a) 30 wt.%, (b) 40 wt.%, and (c) 50 wt.%. Corresponding pore size distributions are shown in (a_1), (b_1), and (c_1), illustrating an increase in pore size with higher PEG1K content.

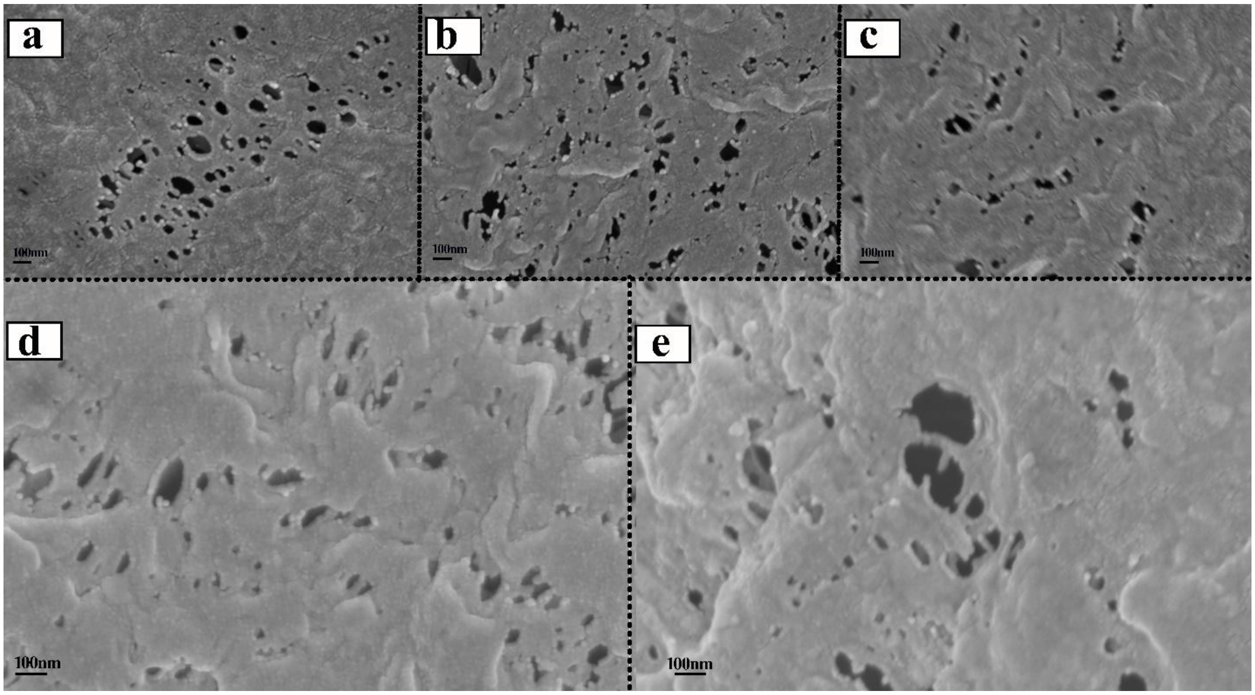

Figure S3. This figure shows the surface pores of the membranes, (a)-(e) denoting the surface pores of 20PVDF30PEG1K, 20PVDF40PEG1K, 20PVDF50PEG1K, 20PVDF, and the Multilayered membrane, respectively.

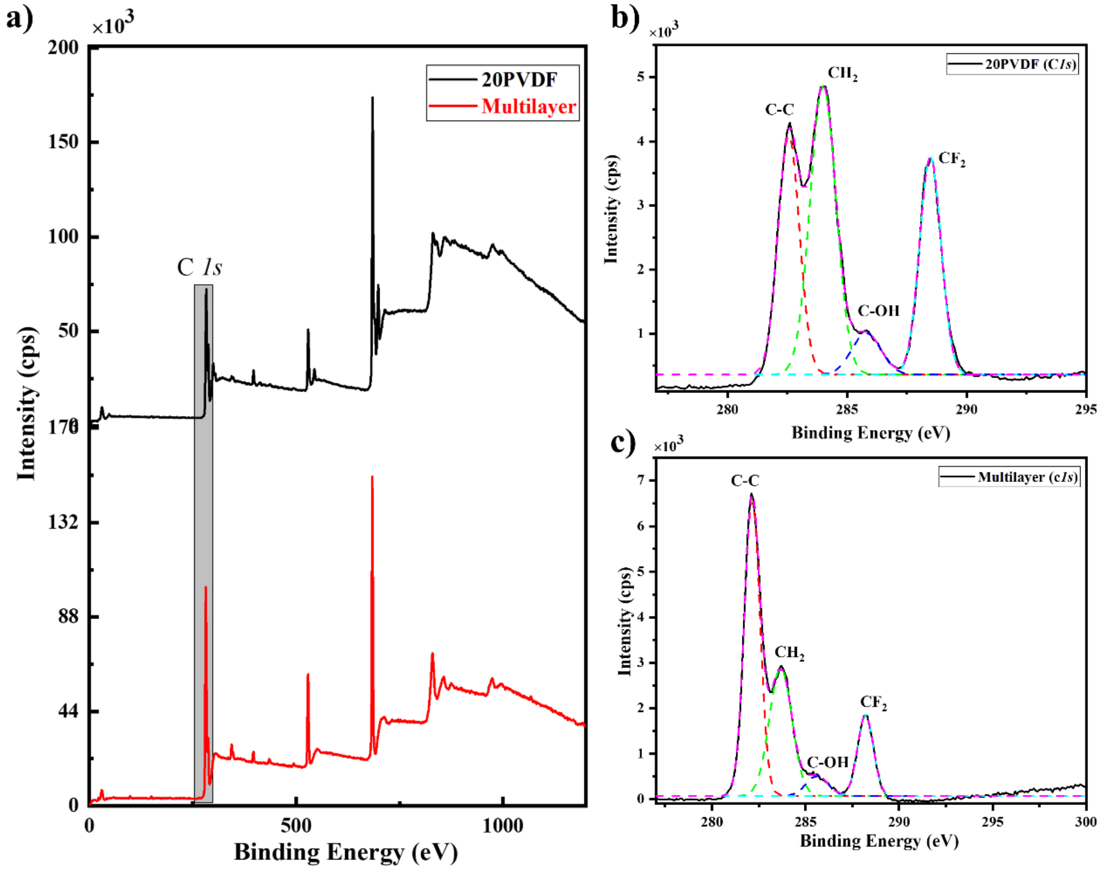

Figure S4. (a) XPS spectra of PVDF and Multilayer membranes, (b), (c) C 1s spectra of PVDF and Multilayer respectively.

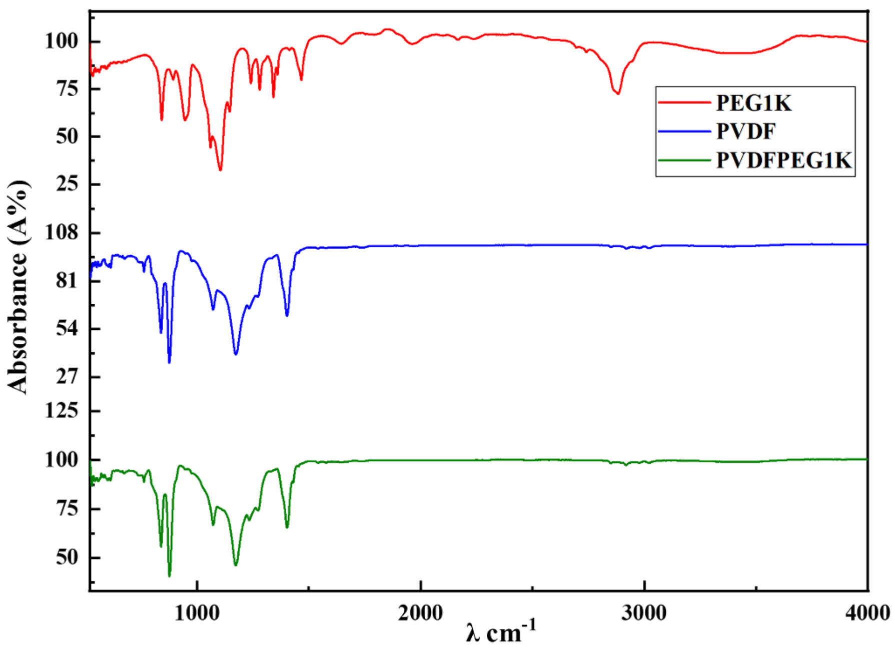

Figure S5. FTIR Absorbance spectra of PEG1K, PVDF, and PVDFPEG1K.

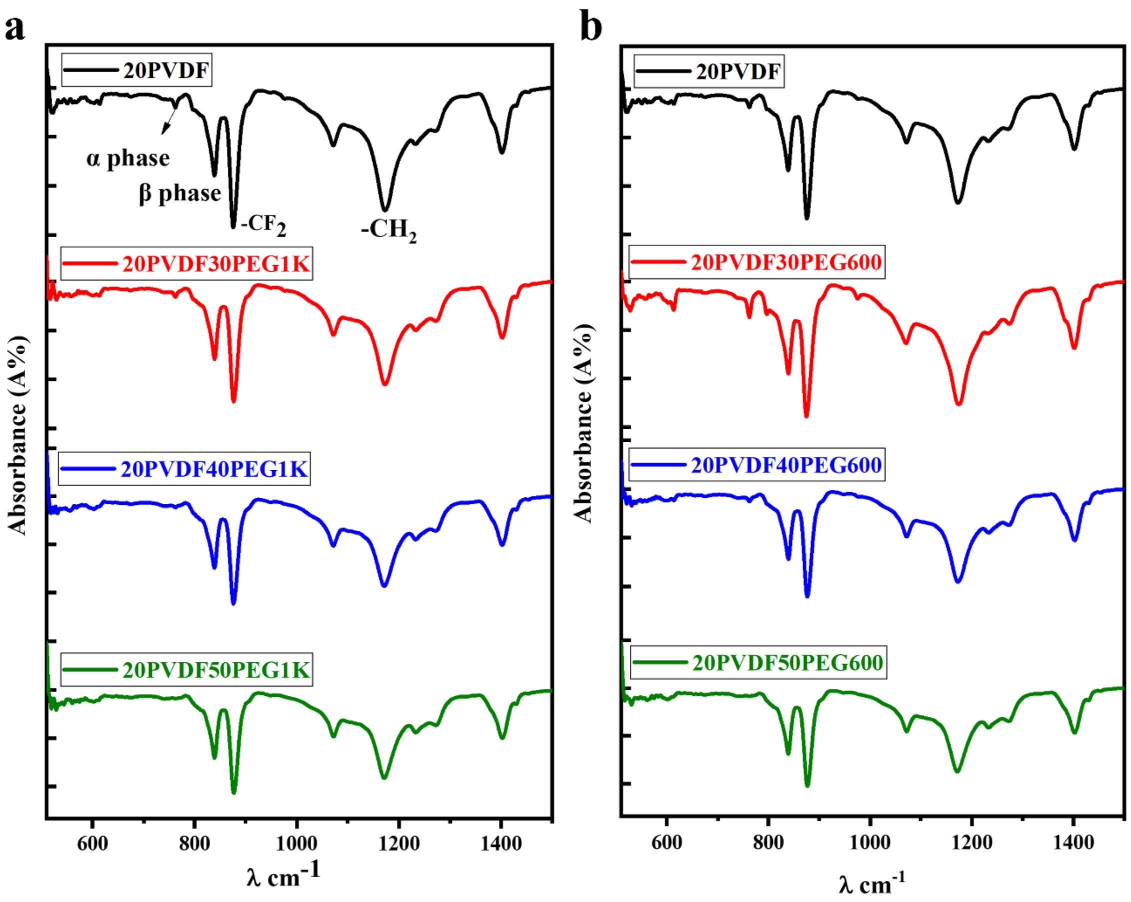

Figure S6. FTIR absorbance spectra with (a): PVDF and PVDF-PEG1K blends and (b) PVDF and PVDF-PEG600 blends spectra.

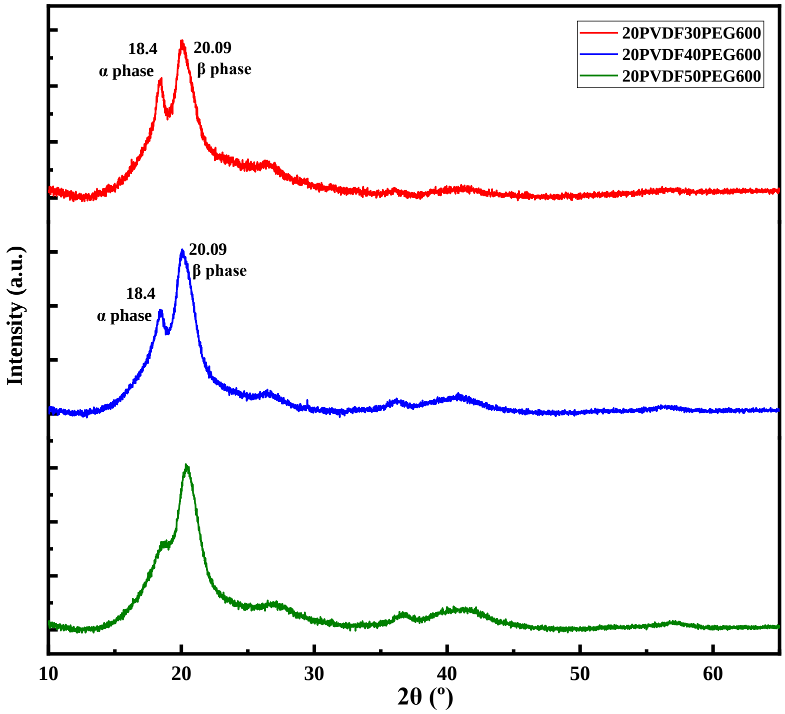

Figure S7. XRD diffractogram of different PVDF-PEG 600 blends.

4. Membrane Properties

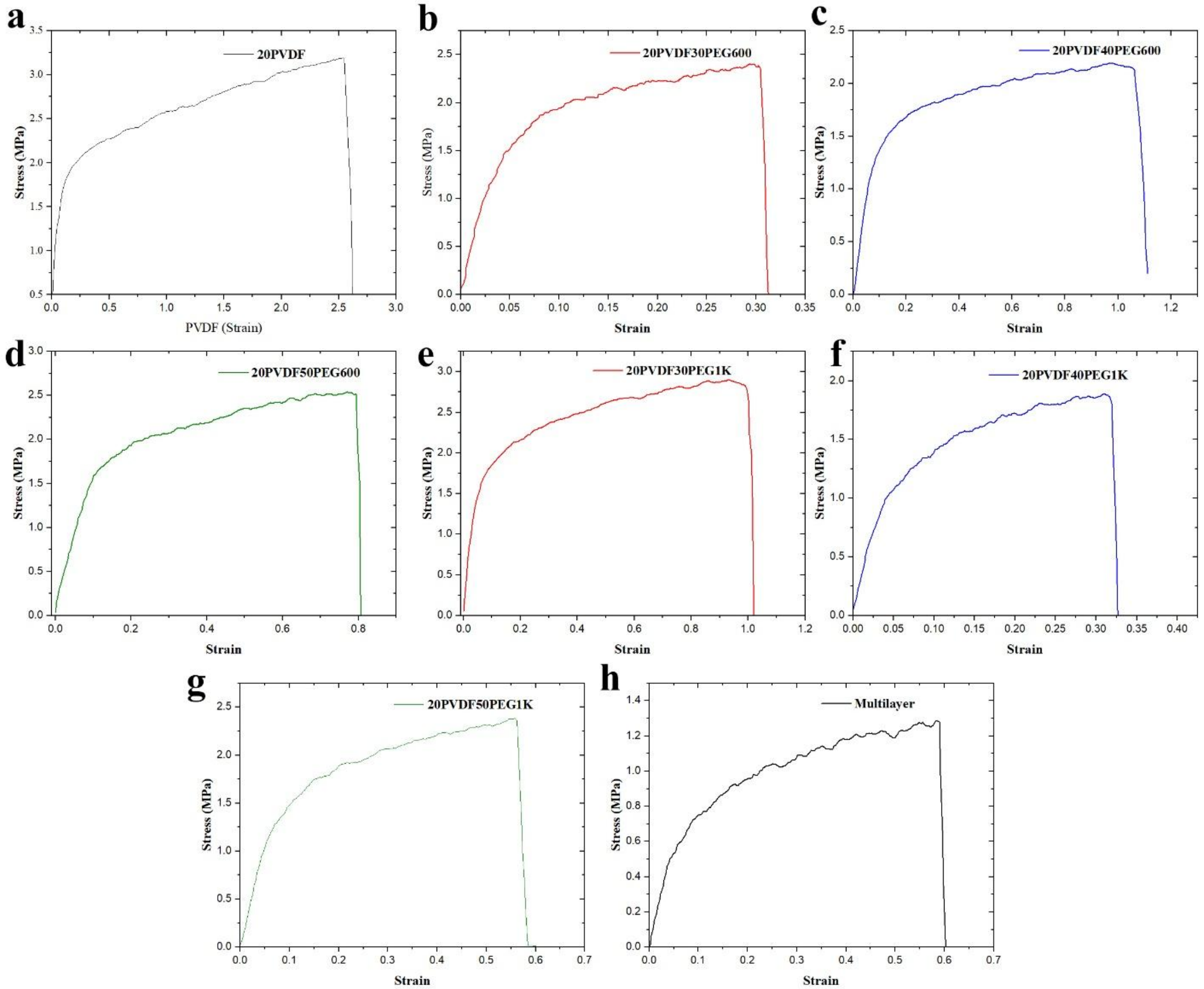

Figure S8. (a)-(h) Stress-strain curves illustrating the mechanical behavior of the various 3D-printed membranes, providing insights into mechanical characteristics under applied stress.

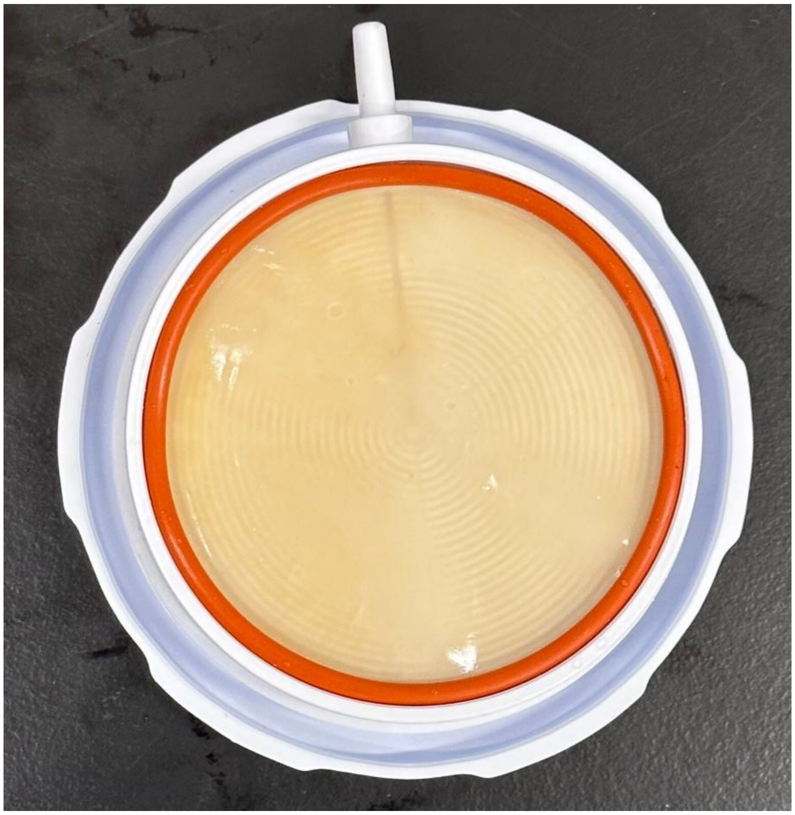

Figure S9. Image of MHM on the stirrer cell after 3 cycles of continuous filtration.

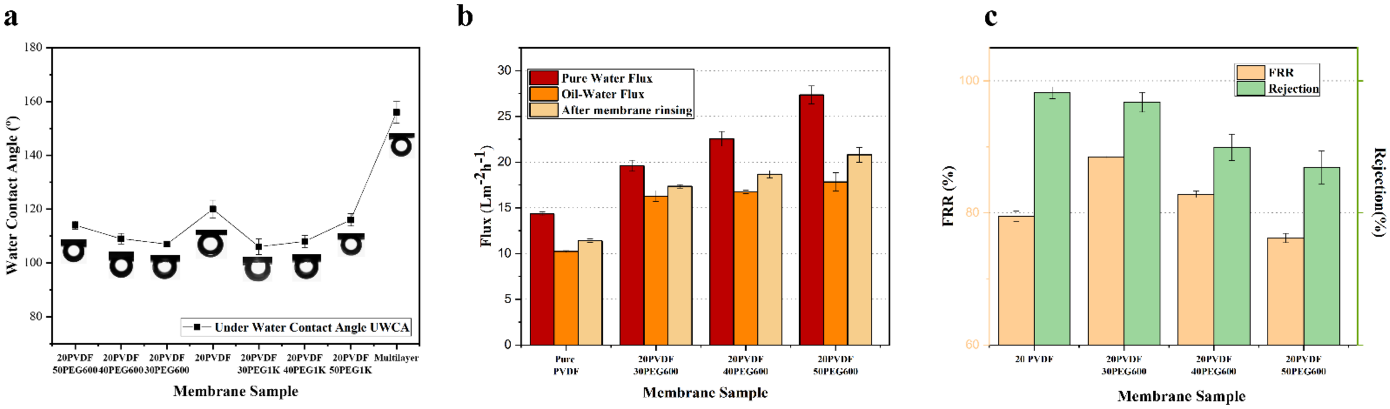

Figure S10. Characteristics and performance metrics of PVDF-PEG600 blend membranes: (a) Underwater contact Angle of different printed membranes. (b) Pure water flux before and after oil-water filtration, demonstrating the membrane's durability and filtration capability; (c) Oil rejection percentage from the oil-water solution and membrane reusability, indicated by the flux recovery ratio, highlighting consistent performance across multiple filtration cycles.

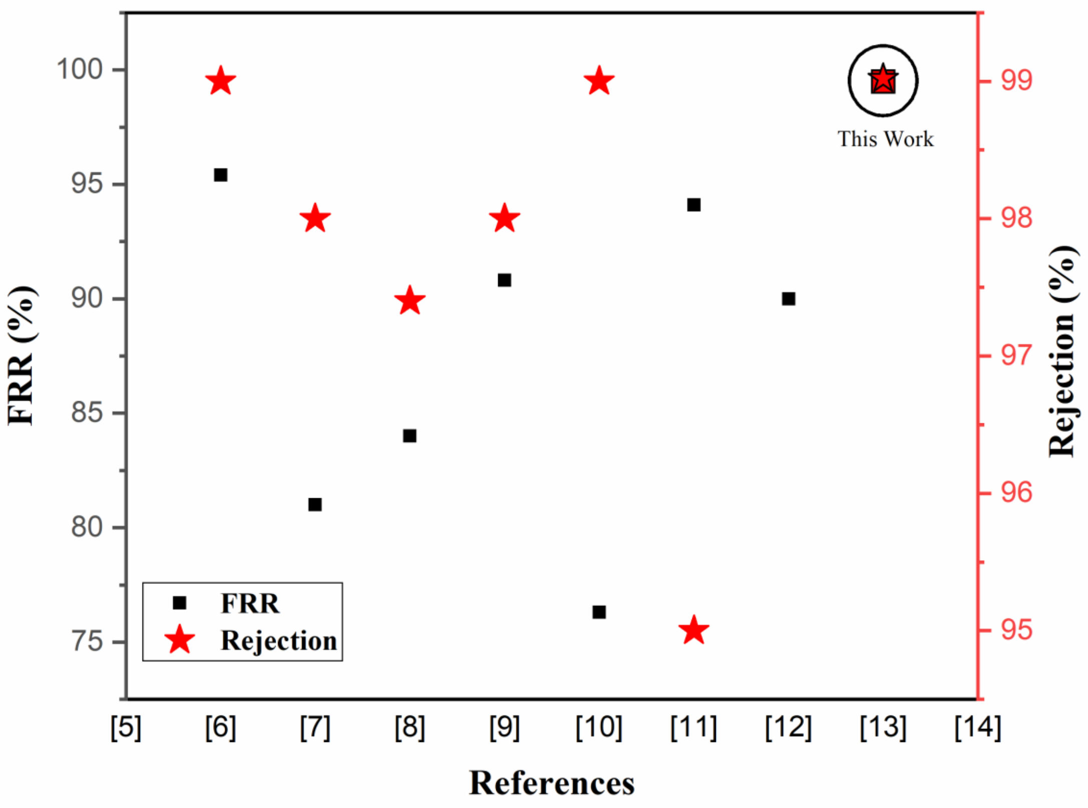

Figure S11. The graph shows the comparison of flux recovery ratio (FRR %) and rejection % from polymer membranes prepared using different techniques highlighting the superior performance of MHMs.

Reference:

(1) Soo, A.; Ali, S. M.; Shon, H. K. 3D Printing for Membrane Desalination: Challenges and Future Prospects. *Desalination* **2021**, *520*, 115366.

(2) Balogun, H. A.; Sulaiman, R.; Marzouk, S. S.; Giwa, A.; Hasan, S. W. 3D Printing and Surface Imprinting Technologies for Water Treatment: A Review. *Journal of Water Process Engineering* **2019**, *31*, 100786.

(3) Ray, S. S.; Dommati, H.; Wang, J.-C.; Chen, S.-S. Solvent Based Slurry Stereolithography 3D Printed Hydrophilic Ceramic Membrane for Ultrafiltration Application. *Ceram Int* **2020**, *46* (8, Part B), 12480–12488.

(4) Yin, G.; He, Q.; Zhou, X.; Wu, Y.; Li, H.; Yu, M. Printing Ionic Polymer Metal Composite Actuators by Fused Deposition Modeling Technology. *Int J Smart Nano Mater* **2021**, *12* (2), 218–231.

(5) Yuan, S.; Strobbe, D.; Kruth, J.-P.; Van Puyvelde, P.; Van der Bruggen, B. Production of Polyamide-12 Membranes for Microfiltration through Selective Laser Sintering. *J Memb Sci* **2017**, *525*, 157–162.

(6) Zhao, J.; Liu, H.; Xue, P.; Tian, S.; Lv, Z.; Wang, R.; Lv, X.; Sun, S. High-Performance PVDF Water Treatment Membrane Based on IL-Na+MMT for Simultaneous Removal of Dyes and Oil-Water Emulsions. *J Environ Chem Eng* **2023**, *11* (1), 109093.

(7) Huang, X.; Wang, W.; Liu, Y.; Wang, H.; Zhang, Z.; Fan, W.; Li, L. Treatment of Oily Waste Water by PVP Grafted PVDF Ultrafiltration Membranes. *Chemical Engineering Journal* **2015**, *273*, 421–429.

(8) Zhao, S.; Wang, Z.; Wei, X.; Zhao, B.; Wang, J.; Yang, S.; Wang, S. Performance Improvement of Polysulfone Ultrafiltration Membrane Using Well-Dispersed Polyaniline–Poly(Vinylpyrrolidone) Nanocomposite as the Additive. *Ind Eng Chem Res* **2012**, *51* (12), 4661–4672.

(9) Vatanpour, V.; Madaeni, S. S.; Khataee, A. R.; Salehi, E.; Zinadini, S.; Monfared, H. A. TiO2 Embedded Mixed Matrix PES Nanocomposite Membranes: Influence of Different Sizes and Types of Nanoparticles on Antifouling and Performance. *Desalination* **2012**, *292*, 19–29.

(10) Teli, S. B.; Molina, S.; Calvo, E. G.; Lozano, A. E.; de Abajo, J. Preparation, Characterization and Antifouling Property of Polyethersulfone–PANI/PMA Ultrafiltration Membranes. *Desalination* **2012**, *299*, 113–122.

(11) Zirehpour, A.; Jahanshahi, M.; Rahimpour, A. Unique Membrane Process Integration for Olive Oil Mill Wastewater Purification. *Sep Purif Technol* **2012**, *96*, 124–131.

(12) Kim, D.-G.; Kang, H.; Han, S.; Lee, J.-C. The Increase of Antifouling Properties of Ultrafiltration Membrane Coated by Star-Shaped Polymers. *J Mater Chem* **2012**, *22* (17), 8654–8661.
